# Epidemic Reconstruction in a Phylogenetics Framework: Transmission Trees as Partitions of the Node Set

**DOI:** 10.1371/journal.pcbi.1004613

**Published:** 2015-12-30

**Authors:** Matthew Hall, Mark Woolhouse, Andrew Rambaut

**Affiliations:** 1 Institute of Evolutionary Biology, University of Edinburgh, Edinburgh, United Kingdom; 2 Centre for Immunity, Infection and Evolution, University of Edinburgh, Edinburgh, United Kingdom; 3 Fogarty International Center, National Institutes of Health, Bethesda, Maryland, United States of America; Ecole Polytechnique Federale de Lausanne, SWITZERLAND

## Abstract

The use of genetic data to reconstruct the transmission tree of infectious disease epidemics and outbreaks has been the subject of an increasing number of studies, but previous approaches have usually either made assumptions that are not fully compatible with phylogenetic inference, or, where they have based inference on a phylogeny, have employed a procedure that requires this tree to be fixed. At the same time, the coalescent-based models of the pathogen population that are employed in the methods usually used for time-resolved phylogeny reconstruction are a considerable simplification of epidemic process, as they assume that pathogen lineages mix freely. Here, we contribute a new method that is simultaneously a phylogeny reconstruction method for isolates taken from an epidemic, and a procedure for transmission tree reconstruction. We observe that, if one or more samples is taken from each host in an epidemic or outbreak and these are used to build a phylogeny, a transmission tree is equivalent to a partition of the set of nodes of this phylogeny, such that each partition element is a set of nodes that is connected in the full tree and contains all the tips corresponding to samples taken from one and only one host. We then implement a Monte Carlo Markov Chain (MCMC) procedure for simultaneous sampling from the spaces of both trees, utilising a newly-designed set of phylogenetic tree proposals that also respect node partitions. We calculate the posterior probability of these partitioned trees based on a model that acknowledges the population structure of an epidemic by employing an individual-based disease transmission model and a coalescent process taking place within each host. We demonstrate our method, first using simulated data, and then with sequences taken from the H7N7 avian influenza outbreak that occurred in the Netherlands in 2003. We show that it is superior to established coalescent methods for reconstructing the topology and node heights of the phylogeny and performs well for transmission tree reconstruction when the phylogeny is well-resolved by the genetic data, but caution that this will often not be the case in practice and that existing genetic and epidemiological data should be used to configure such analyses whenever possible. This method is available for use by the research community as part of BEAST, one of the most widely-used packages for reconstruction of dated phylogenies.

## Author Summary

With sequence data becoming available in increasing high volumes and at decreasing costs, there has been substantial recent interest in the possibility of using pathogen genome sequences as a means to retrace the spread of disease amongst the infected hosts in an epidemic. While several such methods exist, many of them are not fully compatible with phylogenetic inference, which is the most commonly-used methodology for exploring the ancestry of the isolates represented by a set of sequences. Procedures using phylogenetics as a basis have either taken a single, fixed phylogenetic tree as input, or have been quite narrow in scope and not available in any current package for general use. For their part, standard phylogenetic methods usually assume a model of the pathogen population that is overly simplistic for the situation in an epidemic. Here, we bridge the gap by introducing a new, highly flexible method, implemented in the publicly-available BEAST package, which simultaneously reconstructs the transmission history of an epidemic and the phylogeny for samples taken from it. We apply the procedure to simulated data and to sequences from the 2003 H7N7 avian influenza outbreak in the Netherlands.

## Introduction

The increasing availability of faster and cheaper sequencing technologies is making it possible to acquire genetic data on the pathogens involved in outbreaks and epidemics at a very fine resolution. It is likely that in future outbreaks where most or all clinical cases can be identified, pathogen nucleotide sequences will be available from each one as a matter of course. Identification of a high proportion of cases is plausible in several scenarios, such as agricultural outbreaks, where the infected unit will usually be taken to be the farm and considerable government resources will be employed to identify every one, HIV, where almost all infected individuals will eventually seek treatment, and epidemics involving a population that can be closely monitored, such as those occurring in hospitals or prisons. The prospect of acquiring complete or nearly complete sequence datasets from an outbreak naturally suggests the possibility that genetic data could be used to reconstruct the transmission tree, determining which infected host or premises infected which others. Such a procedure would be of value in epidemiological investigations, with genetic data providing a means to complement traditional methods of contact-tracing.

There has been considerable recent work in the development of computational methods to perform analyses of this sort. Early papers inferred links based on pairwise comparisons between isolate sequences [1–4], sometimes combined with epidemiological data, but without explicitly modelling the mutation process. More recent work has instead employed a phylodynamic framework, in which inference is performed using a combination of epidemiological and evolutionary models [5–12]. A Bayesian Markov Chain Monte Carlo (MCMC) approach has almost always been used, as the probability spaces involved are of very high dimension and mathematically complicated.

The most frequent approach has been to start with a model of transmission and attach a mutation model to it, making simplifications that link the evolutionary process with host-to-host transmission events. These simplifications often violate some of the basic principles of phylogenetic inference. Jombart el al. [4, 9] treat mutation as a consequence of transmission, with none occurring within-host, whereas the work of Morelli et al [7], extended by Mollentze et al [11], while allowing for within-host mutation, still only allows a single pathogen lineage to exist within each host at any given time. Such simplifications may be reasonable to make when analysing an epidemic. However, as phylogenetic analysis is the most commonly-used tool for investigating of the history of pathogen lineages, there is scope for the development of methods which are fully compatible with it.

Also implicit within these assumptions of no within-host genetic diversity is another assumption, which is that branching times in the phylogeny and transmission events coincide; this is shared with a number of phylodynamic methods for analysis of less well-sampled datasets [13–17]. Effectively, the transmission tree and phylogeny are taken to be the same entity. Previous studies have shown that this assumption can be problematic [8, 10]. Two pathogen lineages coexisting in a host can share a common ancestor immediately after infection (or even before), but the first transmission from that host to another is unlikely to occur until several days afterwards. An error of several days is of some significance in investigating an infectious disease emergency.

Some methods do acknowledge that the phylogenetic and transmission trees are separate, although related, entities. An exploration of this was performed by Ypma et al. [8], who linked up individual within-host phylogenies according to a transmission tree structure to build a single tree describing the history of the pathogen lineages for an entire epidemic. Other papers have noted that, instead of dealing with multiple phylogenies, a transmission history can be reconstructed by augmenting the internal nodes of a single tree for samples taken from the epidemic with information about the host in which the corresponding lineage was located. This would be a preferable approach in general because it is much more compatible with existing computational methods for phylogeny reconstruction which estimate only a single tree. Cottam et al. [5] were the first to identify this, and it was revisited and refined by Didelot et al. [10].

These two studies, however, were constrained by the lack of a method to co-estimate the complete phylogeny simultaneously with its node labels; they have instead employed a “two-step” procedure, using a fixed tree pre-generated by a standard phylogenetic method. This approach has two problems. Firstly, it will ignore any uncertainty in estimates of the phylogeny. If a Bayesian phylogeny reconstruction method is used, this can be mitigated by using the same method on each one of a sample of trees drawn from the posterior distribution, but at the cost of greater computational time. Secondly, the method used to construct such a fixed tree will often have made assumptions about the structure of population of pathogens or infected hosts that is inconsistent with that of an epidemic. Standard analyses for estimation of time-resolved phylogenies will assume that, all lineages are part of a single, freely mixing population, with the probability of a tree calculated based on the assumption that it was generated by a coalescent process in this population. The result is that phylogenies may display features that are not epidemiologically plausible. For example, even for the fastest-evolving RNA viruses it remains true that many sequences collected over the short timescale of an epidemic will be identical [18]. If this is the case for two isolates, they are likely to form a “cherry” in the reconstructed phylogeny whose time of most recent common ancestor (TMRCA) can take values very close to the sampling time of the earlier isolate, because in a panmictic population, there is no reason to rule this out. In an epidemic situation where each sample is taken from a different host, we know that this is impossible, as there must have been at least one infection event since that TMRCA, and in the time from infection to sampling, a host will have gone through an incubation period and probably also a non-negligible period from manifestation of symptoms to sampling. If a single tree with these short terminal branch lengths is then used to estimate epidemiological parameters, estimates of times from infection to sampling are unlikely to be reliable.

Phylogenetic inference, too, would benefit from a more realistic population model for data from epidemics than the free mixing that is assumed in the standard coalescent-based methods. Much more sophisticated models, designed specifically with epidemics in mind, exist in the field of mathematical epidemiology. Of particular interest are those [19–21] that treat each infected host or premises as an individual entity rather than the member of a compartment, as this aligns closely with phylogenetics, where each isolate must come from one particular host, and allows inference that uses detailed epidemiological data, which can be acquired at the same time that a pathogen sample is taken for sequencing.

Our contribution here is threefold. Firstly, we provide a more rigorous mathematical definition of the correspondence identified by Didelot et al. [10] between an annotation of the internal nodes of a phylogeny with host data and a transmission tree. Secondly, we provide a full, flexible, Bayesian MCMC framework for “one-step”, simultaneous estimation of transmission trees and phylogenies, which uses a model of the pathogen population that is consistent with host-to-host transmission during an epidemic, and can make use of relevant epidemiological data. Thirdly, as our method is fully integrated into the existing phylogenetics application BEAST [22], it provides a freely-available implementation of a method of this type for use by the research community, as well as platform for future development that has access to all the models and methods that are already implemented in that package.

## Methods

### Transmission trees as partitions of the set of nodes of a phylogeny

We suppose that during an infectious disease outbreak or epidemic that infected *N* different units (be they infected organisms or infected premises—we use the word “host” in this section to avoid ambiguity), each of the *N* underwent one or more examinations which detected whether it was infected or not. Hosts that were found to be infected at an examination provided a pathogen isolate from which was obtained a nucleotide sequence (a positive examination); hosts that were not provided nothing (a negative examination). An examination could produce at most one sequence but multiple examinations could be performed simultaneously. We assume that each host experienced at least one positive examination, so we are aware of all infections. The nucleotide sequences resulting from these examinations, together with information on negative examinations, forms our dataset *D*. It may be that there are known hosts in the epidemic for which no sequence is actually available; in these cases it is obvious that if an examination was made at a time at which we know infection was present, it would have been positive and have provided a sequence, so we declare that such an examination occurred but produced a noninformative sequence (i.e. consisting entirely of the code “N” representing “any nucleotide”). As a result we have *M* pathogen sequences with *N* ≤ *M*. We denote the set of examination times by **T** ^exam^. We also assume no superinfection or reinfection, and that transmission is a complete bottleneck; only one genetic variant is passed from an infectious host to a newly infected one.

A phylogeny G, with branch lengths in units of time, describes the ancestral relationship between the sequences from all positive examinations. The “height” of nodes in this full tree is defined in backwards time relative to the time at which the last positive examination was made. If **A** is the complete set of *N* hosts, a transmission tree in our terminology is a rooted tree with *N* nodes labelled with the elements of **A**. The root node of such a tree is labelled with the first host in the outbreak and edges indicate infections. They do not include information on timings and consist solely of a description of which host infected which others. As such, the tree can be regarded as a map taking each host to its infector, or to nothing if it is the index host.

**Fig 1:**
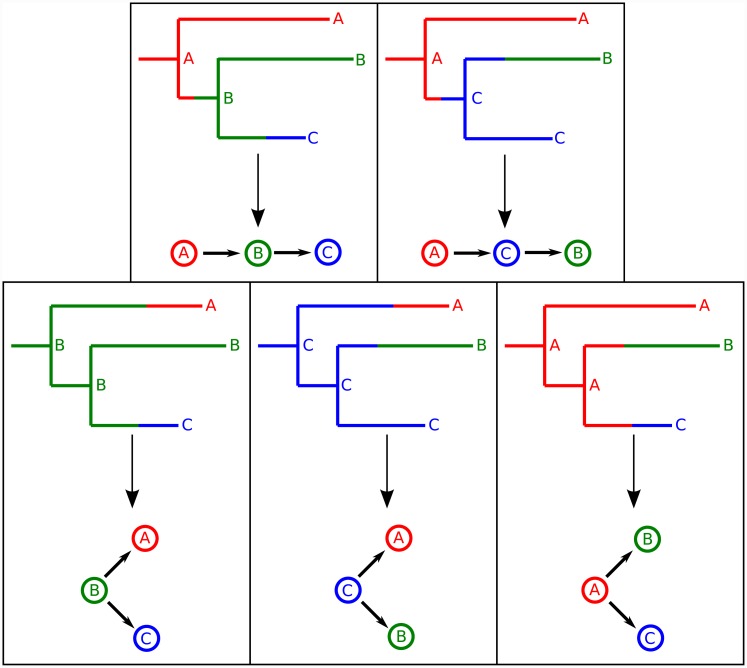
The five possible transmission tree structures of a phylogenetic tree with three tips, depicted as partitions of the nodes of a phylogeny (above) and as directed graphs amongst the hosts A, B and C (below).

Didelot et al. [10], traced the spread of a pathogen amongst hosts by annotating each internal node of a phylogeny with the host that the corresponding lineage was present in. We use the same principle. Formally, there is a correspondence between possible transmission trees and ways in which the internal nodes of G can be partitioned into subsets subject to two rules: that, for each such subset, the subgraph of G consisting of all the nodes in the subset and all edges connecting them (this is called the subgraph induced by the subset) is connected, and that each subset contains all the tips corresponding to the isolates taken from one and only one host. The annotation then associates each node with the host from which the tips in its subset were taken from. Fig 1 shows this correspondence for the simple case where there are three hosts in the epidemic with one isolate taken from each. In S1 Text we give a more extensive mathematical treatment of this correspondence, demonstrating that it is one to one if the phylogeny is fixed, that not every transmission tree arises as a partition of the nodes of such a fixed phylogeny if there are more than two hosts, but that every one does arise as a partition of the nodes of some phylogeny.

In such a partitioned phylogeny, infection events occur on branches joining nodes which have been annotated with different hosts. The host that the parent node is annotated with infects the host that the child node is annotated with. If the latter host is *a*_*i*_, we call this branch the infection branch of *a*_*i*_. The infection branch of the host that was the index case in the epidemic is the root branch of the phylogeny, which we, in contrast to most phylogenetic methods, give a finite length. The timings of the two nodes joined by this branch constrain the infection time of *a*_*i*_, but the partition does not exactly specify it. Assuming that infection times and times of coalescence of lineages cannot exactly coincide, to fully describe the epidemic we introduce, for each host *a*_*i*_, a parameter *q*_*i*_ between 0 and 1 such that if the infection branch of *a*_*i*_ starts at *t*_1_ and ends at *t*_2_, then *a*_*i*_ was infected at $t_{i}^{\text{inf}}=t_{1}+q_{i}\left(t_{2}-t_{1}\right)$.

### MCMC procedure

As many transmission histories cannot be reconstructed by partitioning the nodes of a single phylogeny, a reconstruction procedure that is able to fully explore the space of transmission trees cannot simply take a fixed tree as input. Instead, it must explore the full space of phylogenetic trees as well. The most common methods for estimation of time-resolved phylogenies involve the use of Bayesian MCMC to sample from the probability distribution of phylogenetic trees given the available sequence data. If the data is as outlined above, such procedures can be extended to simultaneously sample from the probability distribution of reconstructed epidemics if each sampled tree is augmented with a partition of its internal nodes as well as parameters determining the exact times of infection of each host. We have implemented this procedure in the package BEAST [22]. Because of the special requirements of this type of augmentation, the standard MCMC moves on a phylogenetic tree topology are unsuitable as they will generally not make modifications that respect the rules of the node partitions. Instead, a specialised set moves have been devised to alter the phylogeny and partition in such a way that the transmission tree structure is maintained, which we now describe.

#### Infection branch operator

**Fig 2:**
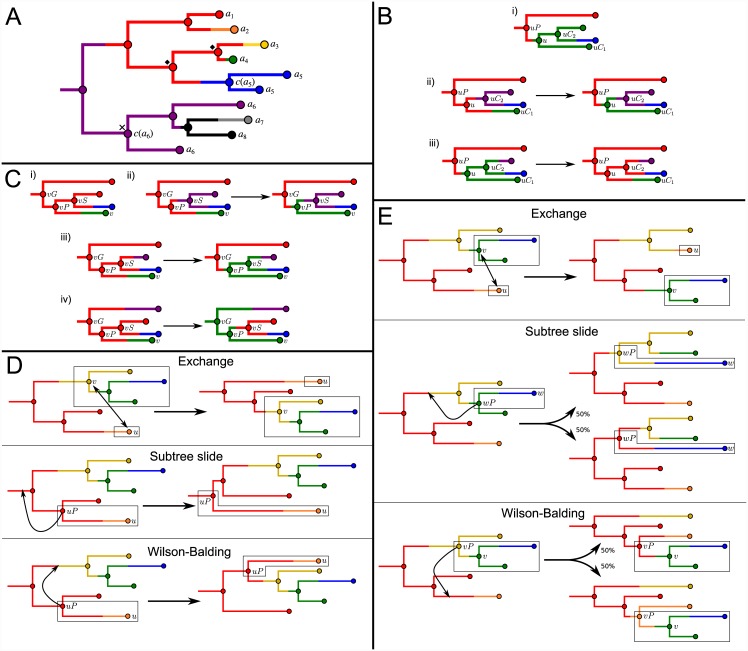
Illustrations of partitioned phylogenies and MCMC proposals modifying them. Nodes in all cases are coloured by the partition element containing them. (A) An example partitioned phylogeny. Tips are labelled by the hosts that the isolates corresponding to them were taken from. Where more than one isolate is taken from a host *a*_*i*_, *c*(*a*_*i*_) is labelled; in all other cases *c*(*a*_*i*_) is the single tip corresponding to an isolate taken from that host. Black diamonds designate nodes that are not ancestral under the partition. The hosts *a*_7_ and *a*_8_ are root-blocked by *a*_6_ due to the position of *c*(*a*_6_) (black cross). (B) The downward infection branch move. The move attempts to move the node *u* from the green partition element to the red (which already contains its parent *uP*). In i), the move is impossible because *u* is the MRCA node of the tips in the green element. In ii), it can be done with no further modifications required to obey the rules. In iii), the node *uC*_2_, which is not ancestral under the initial partition, must also be moved to the red element so the result obeys the rules. (C) The upward infection branch move. The move attempts to move the node *vP* from the red partition element to the green (which already contains its child *v*). In i), the move is impossible because *vP* is ancestral under the partition and the host represented by the green element is root-blocked by the host represented by the red. In ii), it can be done with no further modifications required to obey the rules. In iii), the node *vS*, which is not ancestral under the initial partition, must also be moved to the green element, and in iv) the node *vG* must be because *vS* is ancestral. (D) The type A phylogeny moves. The exchange move exchanges the nodes *u* and *v*; the subtree slide and Wilson-Balding moves change the position of the node *u* and its parent *uP*. (E) The type B phylogeny moves. The exchange move exchanges the nodes *u* and *v*; the subtree slide move the node *w* and its parent *wP*, and the Wilson-Balding the node *v* and its parent *vP*. After the latter two moves the transplanted parent node is randomly assigned to one of two new partition elements with equal probability.

This operator changes the partition of the phylogeny G while keeping G itself fixed. We first need to introduce some terminology. If P is a partition of the nodes of G as described above, and *u* is a node of G, let $d_{P}\left(u\right)$ be the host from which the tips in the same element of P (remembering that elements of P are subsets of the set of nodes of G) as *u* were sampled. This is in fact the host in which the pathogen lineage at *u* was present. Say that an internal node *u* of G is ancestral under P if it is an ancestor of at least one tip of G that is a member of the same element of P as itself (a tip that is associated with the same host as itself). For a host *a*_*i*_, let *c*(*a*_*i*_) be the most recent common ancestor node of all the tips in G that correspond to samples taken from *a*_*i*_. (If there is only one such tip, *c*(*a*_*i*_) is just that tip.) Observe that $d_{P}\circ c\left(a_{i}\right)$ must be *a*_*i*_ itself, because if it is not then the subgraph induced by all nodes mapping to *a*_*i*_ under $d_{P}$ will not be connected. Say that a host *a*_*i*_ is root-blocked by a host *a*_*j*_ if *c*(*a*_*j*_) is an ancestor of *c*(*a*_*i*_). The reason for this nomenclature is that if this is true, the root *r* of G can never have $d_{P}\left(r\right)=a_{i}$ because if the nodes of a connected subgraph of G include both *r* and *c*(*a*_*i*_) then they must also include *c*(*a*_*j*_), and this is untrue because $d_{P}\circ c\left(a_{j}\right)=a_{j}$. See subfigure A in Fig 2 for an illustration of these concepts.

The move operates by first randomly picking a host *a*_*i*_ that is not the index host, and finding its infection branch. If this ends in *u* and begins in *uP*, then $d_{P}\left(u\right)$ and $d_{P}\left(uP\right)$ are different hosts. Suppose *p*_1_ is the partition element containing *u* and *p*_2_ contains *uP*. We want to produce a new partition $P^{\prime }$ in which both *u* and *uP* are in either *p*_1_ or *p*_2_, adjusting the membership of all elements so that the subgraphs remain connected. This pushes the infection branch of *a*_*i*_ up or down the tree (in our terminology “up” is towards the root). This is not always possible in both directions, but if it is, then we select upwards or downwards each with probability 0.5. If neither is possible then the move fails.

The downward move, which moves the infection branch of *a*_*i*_ towards the tips and puts *u* into *p*_2_, is impossible if *u* = *c*(*a*_*i*_) as removing the MRCA of all the tips in a subtree will always disconnect that subtree. This also prevents the move from changing which partition element any tip belongs to, because *u* is a tip then *u* = *c*(*a*_*i*_). If *u* ≠ *c*(*a*_*i*_), then let *uC*_1_ and *uC*_2_ be the two children of *u*. If $d_{P}\left(uC_{1}\right)=d_{P}\left(uC_{2}\right)$ then the reassignment of *u* to *p*_2_ will have disconnected the subgraph induced by *p*_1_. But we know that all the tips that were contained in *p*_1_ must be descended from only one of *uC*_1_ and *uC*_2_ (or else *u* = *c*(*a*_*i*_)); in other words only one of these nodes is ancestral under P. Without loss of generality say it is *uC*_1_. We move *uC*_2_ and all descendants of it that are also in *p*_1_ to *p*_2_ as well. This move is depicted in Fig 2B.

The upward move works in the opposite way; *uP* is moved to *p*_1_. If $d_{P}\left(uP\right)=a_{j}$, then this is impossible if *a*_*i*_ is root-blocked by *a*_*j*_ and *uP* is ancestral under P. Suppose *uG* is *uP*’s parent (which may not exist if *uP* is the root) and *uS* its other child. If either *uG* does not exist, or *uP* and *uS* are in different elements of P, then nothing further is required. Otherwise, changing the partition element containing *uP* disconnects the subgraph induced by *p*_2_. Only one of its components can contain any tips, because if both did, *uP* would be ancestral under P since it would be the ancestor of the tips in one component, and *a*_*i*_ would be root-blocked by *a*_*j*_. If *uP* is ancestral under P then the component containing *uS* contains them; if it is not then the one containing *uG* does. We complete the move by moving all nodes in the component with no tips to *p*_2_ are well. This move is depicted in Fig 2C.

We then note that:

- The downward move on *u* is reversed by the upward move on the child *uC*_1_ of *u* that is ancestral under P. The Hastings ratio is 1 multiplied by 2 if $uC_{1}=c\circ d_{P^{\prime }}\left(uC_{1}\right)$ and by 0.5 if *u* is ancestral under P and $d_{P}\left(u\right)$ is root-blocked by $d_{P}\left(uP\right)$.
- If *uP* is not ancestral under P, then the upward move on *u* is reversed by the downward move on *uP*. The Hastings ratio is 1 multiplied by 0.5 if $u=c\circ d_{P}\left(u\right)$ and by 2 if *uG* is ancestral under P and $d_{P^{\prime }}\left(uP\right)$ is root-blocked by $d_{P^{\prime }}\left(uG\right)$.
- If *uP* is ancestral under P, and the upward move on *u* is possible, then it is reversed by the upward move on its sibling *uS*. The Hastings ratio is 1 multiplied by 0.5 if $u=c\circ d_{P}\left(u\right)$ and then by 2 if $uS=c\circ d_{P^{\prime }}\left(uS\right)$.

#### Phylogeny operators

BEAST in its default configuration uses three types of phylogeny operator: exchange [23], subtree slide [24], and Wilson-Balding [25]. All three have been modified to produce two special cases which respect node partitions. The “type A” operators do not change the transmission tree, whereas the “type B” moves simultaneously rearrange both trees. For brevity we sketch these here; a full treatment can be found in S1 Text, in which we also show that the Markov chain is irreducible. Figs 2D and 2E depict examples of the modifications made to a partitioned tree by the type A and type B moves respectively.

The “wide” version of the standard exchange operator randomly selects two nodes in the phylogeny that are not siblings or the root, and whose heights and parent heights are such that swapping their parents would not lead to a situation where a node is lower in the tree than its child, and does that swap. Our type A modification randomly selects two nodes that are also not siblings or the root and whose parents are members of the same partition element, and does the same. This preserves the infector of every host (see section S1.3.2.1 in S1 Text for details). The type B version selects instead a random two nodes whose parents are in different partition elements to themselves, and again swaps these parents. If *u* and *v* are the two nodes moved and P is the original partition, then $d_{P}\left(u\right)$ and $d_{P}\left(v\right)$ exchange infectors in the transmission tree (if they were different to start with). The Hastings ratio must be calculated by specifically enumerating the number of possible exchange partners for each node if they were the first of the pair to be selected.

The standard subtree slide operator picks a random node *u*, draws a value *d* from a probability distribution that has support on the whole real line and is symmetric about 0, and moves *u*’s parent *uP* a distance Δ up or down the tree (according to Δ’s sign) along a path connecting the root to the tips; where the move is towards the tips a random branch is chosen at every split encountered. The move fails if *uP* is taken so far down the tree that its height is equal to or smaller than that of *u*. It also may be that *uP* moves so far up the tree that it becomes the root, which is a legal move. The type A modification does the same, but insists that the final position of *uP* is such that it is adjacent to a node in the same partition element as itself. This ensures the transmission tree structure is unchanged. The Hastings ratio must be calculated, again, by enumerating the set of possible origins and destinations. The type B version picks *u* such that *uP* is in a different partition element to itself, then performs the standard move. Afterwards, *uP* is randomly allocated to the partition element containing either its new parent or its new second child with probability 0.5 each. This moves transplants the subtree of the transmission tree rooted at $d_{P}\left(u\right)$ to a new location. The Hastings ratio is the same as for the standard move, save for a trivial modification to take into account the random allocation of *uP* to a new partition element.

The standard Wilson-Balding move picks a random node, and then prunes and reattaches its parent to a random position so long as there is no height conflict. The modifications are largely the same as those for subtree slide; type A can be applied to any node and reattaches its parent in a position adjacent to a node in the same partition element, whereas type B chooses a node with a parent in a different partition element to itself, performs the standard move, then reallocates the parent to a new element. The modifications to the Hastings ratio calculations are also analogous to subtree slide.

Moves are also needed to adjust branch lengths in the phylogeny; these are inherited from BEAST with no modification required.

#### Infection times

None of these these moves change the value of any of the *q*_*i*_ parameters that exactly determine infection times; new values of those are proposed and evaluated separately by draws from a uniform distribution. Nevertheless, changes to either tree may involve modifications of the times of infection of some hosts. For example, the infection branch operator changes the branch on which $d_{P}\left(u\right)$’s infection occurs, so it must change $t_{i}^{\text{inf}}$ even if it does not change *q*_*i*_. Even a move that has no effect on the partition or phylogenetic tree topology, such as a change to branch lengths, may alter the height of the nodes which *a*_*i*_’s infection branch connects, which will also modify $t_{i}^{\text{inf}}$ while *q*_*i*_ remains the same.

### Model description

We assume that the epidemiological and evolutionary processes involved in an epidemic can be described by three models: a stochastic model of infection and between-host transmission dynamics, a deterministic model of the population dynamics of a within-host population of “agents”, and a stochastic model of sequence evolution. Table 1 summarises the notation we will use to describe them in the following paragraphs.

**Table 1:** Description of symbols used in the probability decomposition.

Symbol	Type	Meaning
**T** ^exam^	Background information	Examination times of each host
*L*	Background information	Information defining the relationship between hosts used to define *F* (e.g. spatial locations)
*D*	Data	Results of examinations (sequence data and notes of negative observations)
*b*	Model parameter	Unmodified transmission rate
*ϕ*	Model parameters	Parameters of *F*
*ψ*	Model parameters	Parameters of the population dynamics of the agents within each host
*ρ*	Model parameters	Parameters of distribution of infectious periods
*ω*	Model parameters	Parameters of nucleotide substitution and molecular clock models
G	Latent variable	Phylogenetic tree
N	Latent variable	Transmission tree
**T** ^inf^	Latent variables	Times of infection of each host
**T** ^*trans*^	Latent variables	Times of infectiousness of each host (if different to **T** ^inf^)
**T** ^end^	Latent variables	Times of becoming noninfectious of each host
*F*	Function	Function modifying *b* based on known relationships between hosts

In contrast to the previous work of Didelot et al. [10], whose underlying model of transmission was a compartmental SIR model, we use an individual-based model similar to those employed in previous work on agricultural outbreaks [5–8]. This much more readily allows for the accommodation of host heterogeneity, and makes no assumption of random mixing. We start with a population of susceptible hosts. We may know *a priori* some characteristics that allow us to define relationships between these hosts; if so, call these characteristics *L*. *L* could, for example, be the spatial locations of farms in an agricultural outbreak. The epidemic starts when a single susceptible is infected by an external source. If *a*_*i*_ is a host, $t_{i}^{\text{inf}}$ is its time of infection. It is infectious from $t_{i}^{\text{inf}}$ until a time $t_{i}^{\text{end}}$. The value of $t_{i}^{\text{end}}$ is randomly determined at $t_{i}^{\text{inf}}$, by a draw from a probability distribution with parameters *ρ*. Let **T** ^inf^ be the complete set of infection times and **T** ^end^ the complete set of noninfectiousness times. For now, we assume that a host becomes infectious immediately upon infection; we relax this assumption in a later section. If *a*_*i*_ is infectious and *a*_*j*_ susceptible, *a*_*i*_ inflicts a constant force of infection on *a*_*j*_ given by a rate *b* modified by multiplication by a positive real number *F*(*a*_*i*_, *a*_*j*_), where *F* is a positive function with parameters *ϕ* defining a relationship between *a*_*i*_ and *a*_*j*_ based on the information in *L*. In other words, the time between the infection of *a*_*i*_ and a possible infection of *a*_*j*_ by *a*_*i*_ is drawn from an exponential distribution with mean 1/(*bF*(*a*_*i*_, *a*_*j*_)). If the time drawn is such that *a*_*i*_ was no longer infectious at that point, or if some other infectious host had infected *a*_*j*_ at an earlier time, nothing happens. Otherwise, *a*_*j*_ becomes infected after this time. After $t_{i}^{\text{end}}$, *a*_*i*_ is considered removed and plays no further part in the epidemic.

There are many possible choices for *F*. If we assume no spatial structure or other heterogeneity affecting transmission then we can just take *F*(*a*_*i*_, *a*_*j*_) = 1 for all hosts *a*_*i*_ and *a*_*j*_. Otherwise, it can be based on, for example, geographical distance between sampling sites, a network metric, or shared membership in some risk group. It can also be used to state prior information about the transmission tree structure; if it is known *a priori* that *a*_*i*_ did not infect *a*_*j*_, then *F*(*a*_*i*_, *a*_*j*_) can be set to zero. There is also no requirement that *F* be symmetric.

We assume that each host is examined at least once while it is infected, and that examination does not disturb the course of the infection. Beyond that no concrete assumptions need to be made about the examination process; any number of examinations can be made of any hosts at any time. If examinations are instead restricted so that they only occur at at the point of noninfectiousness of each host, however, there are mathematical advantages, as will be seen.

As in previous work [8, 10] we take the model of the dynamics of the “agents” to be a coalescent process, with parameters *ψ*, amongst lineages in a freely-mixing population within each host. If the hosts are single organisms, the agents will naturally be individual pathogens. If, on the other hand, they are infected locations, they could instead be considered to be infected organisms. In either case, only a very small proportion of the total agent population are represented by lineages in the tree, and the assumption of a low sampling fraction required for use of the coalescent process is satisfied.

The sequence evolution model is of the standard type used in the reconstruction of time-resolved phylogenies [23]. It consists of both a continuous-time Markov chain model of sequence evolution (such as the commonly-used HKY [26] or GTR [27] models) and a molecular clock model. Denote the parameters of both by *ω*. We assume that mutation is a neutral process, and that it occurs independently of the host-to-host transmission structure.

### Bayesian decomposition

In this section we show how the likelihood of a partitioned phylogeny can be calculated using the three models described above. We condition on **T** ^exam^ and *L*. The noninfectiousness times **T** ^end^ are formally considered to be latent variables and could be estimated, but for this paper we assume them to be known and fixed to their actual values, much as Didelot et al. [10] treat removal times. If any or all hosts are known to have remained infectious indefinitely, the corresponding values of **T** ^end^ can be set to the time of analysis. It should be noted that the **T** ^exam^ are not strictly sampling times. They instead represent times at which it is known that hosts were examined, and an infected host would provide a sequence. *D* is the results of these examinations, including the results of negative ones. This formulation allows for some convenient mathematics but has consequences for estimation of the prior distribution (see Discussion). Alternatively, if the data is such that all samples from each host were taken at the same time and the assumption that all hosts ceased to be infected immediately after this time is reasonable, we need not treat **T** ^exam^ in this way and *D* can consist solely of sequence data as it does in standard phylogenetic analyses; see “an alternative approach” below.

Ideally, it should be possible to enumerate all individuals or premises which were susceptible to infection but never experienced it, and *L* should include background information on them. Their never-infected status is assumed *a priori* and since they will never appear in the phylogeny, there is no need to consider examination times for them. For convenience we give these hosts infection and noninfectiousness times whose values are fixed to the time of analysis, as we need to evaluate the probability that they were not infected at any time before the present. Consideration of the never-infected set is necessary for unbiased estimation of *b* and *ϕ*, which should only be interpreted literally if such data is present in the analysis. If it is not, we are actually estimating parameters *b*′ and *ϕ*′, which is what *b* and *ϕ* would be if all susceptibles did experience infection.

The posterior probability we are interested in calculating is $p(G,N,T^{\text{inf}},T^{\text{end}},b,\phi ,\psi ,\rho ,\omega |D,T^{\text{exam}},L)$. By Bayes’ Theorem this is equal to

$$\begin{array}{c} \frac{p\left(D|G,N,T^{\text{inf}},T^{\text{end}},b,\phi ,\psi ,\rho ,\omega ,T^{\text{exam}},L\right)p\left(G,N,T^{\text{inf}},T^{\text{end}},b,\phi ,\psi ,\rho ,\omega |T^{\text{exam}},L\right)}{p(D|T^{\text{exam}},L)}. \end{array}$$

As usual, we need not calculate the denominator if we are uninterested in model comparison as it does not vary. If *D* may contain the results of negative examinations, we must explicitly state that if *D* includes *M* sequences but G has any number of tips other than *M*, then the probability of *D* given G is zero. A G with a different number of tips does not necessarily have zero prior probability, but it does result in zero likelihood for the data, so we need not concern ourself with exploring the posterior probability space of such phylogenies. Given a G with the right number of tips, *D* depends by the assumptions of the mutation model only on G and *ω*, and the likelihood reduces to p(D|G,ω), which can be calculated using the Felsenstein pruning algorithm and the chosen molecular clock model in the normal way [23, 28, 29]. It remains to calculate the prior probability $p(G,N,T^{\text{inf}},T^{\text{end}},b,\phi ,\psi ,\rho ,\omega |T^{\text{exam}},L)$. We decompose this as

$$\begin{array}{ccc} p(G,N,T^{\text{inf}},T^{\text{end}},b,\phi ,\psi ,\rho ,\omega |T^{\text{exam}},L) & = & p(G|N,T^{\text{inf}},T^{\text{end}},b,\phi ,\psi ,\rho ,\omega ,T^{\text{exam}},L)\\ & & \times p(N,T^{\text{inf}},T^{\text{end}}|b,\phi ,\psi ,\rho ,\omega ,T^{\text{exam}},L)\\ & & \times p(b,\phi ,\psi ,\rho ,\omega |T^{\text{exam}},L). \end{array}$$

We make the following assumptions:

- All parameters are independent of *ω*; the mutation process has no bearing on the infection dynamics inside or between hosts.
- The phylogeny G is conditionally independent of *b*, *ϕ*, *ρ*, and *L* given *ψ*, N, **T** ^inf^, **T** ^end^, and **T** ^exam^. The former parameters determine the transmission model and are not relevant if we already know the full transmission tree and its timings.
- The transmission tree N and its event timings **T** ^inf^ and **T** ^end^ are conditionally independent of **T** ^exam^ and *ψ* given *ϕ*, *ρ*, and *L*. The parameters of the within-host model are not relevant to the between-host model and examination is assumed not to disturb the transmission process.
- *b*, *ϕ*, *ψ*, *ρ*, and *ω* are independent of **T** ^exam^, *L* and each other. The parameters determining transmission, within-host growth, infectious periods, and mutation are independent of each other, the examination process, and the exact relationships amongst this set of hosts.

The decomposition is therefore reduced to

$$\begin{array}{ccc} p(G,N,T^{\text{inf}},T^{\text{end}},b,\phi ,\psi ,\rho ,\omega |T^{\text{exam}},L) & = & p(G|N,T^{\text{inf}},T^{\text{end}},\psi ,T^{\text{exam}})\\ & & \times p(N,T^{\text{inf}},T^{\text{end}}|b,\phi ,\rho ,L)\\ & & \times p(b)p(\phi )p(\psi )p(\rho )p(\omega ). \end{array}$$

We first need to calculate $p(G|N,T^{\text{inf}},T^{\text{end}},\psi ,T^{\text{exam}})$. The first observation we make is that the combination of **T** ^inf^, **T** ^end^ and **T** ^exam^ determines which examinations were positive, and that positive examinations correspond to the tips of G. If the number of positive examinations of a given host and the number of tips corresponding to sequences taken from that host differ, then this term must be equal to zero. In theory, we can calculate it for a phylogeny with any number of tips up to the total number of examinations, but in practice we need not if we are sampling from the posterior distribution, as any tree that does not have *M* tips will have zero posterior probability because the likelihood will be zero. So we can assume that G has *M* tips and that no tip date is before the infection date or after the noninfectiousness date of the corresponding host, and merely check that **T** ^inf^, **T** ^end^ and **T** ^exam^ imply *M* positive observations.

If the tip count is correct, we then calculate this probability by extending the procedure outlined by Didelot et al. [10] to allow for the use of any of the standard models of deterministic population growth, and the possibility of host heterogeneity. The latter is accomplished by dividing the set of hosts into categories and assigning a separate demographic model to all of those in each one. Categories can be assigned from known epidemiological data about the hosts; for example, in a livestock disease outbreak, they may reflect the size of farm. Naturally, there is no requirement that there be more than one category. If **c** is such a category, there is a corresponding demographic function $N_{c}:R\to R^{+}$ with parameters *ψ*_**c**_ where *N*_**c**_(*t*) is the product of the effective population size and generation time of the agents at time *t* on a separate backwards timescale in each host. Let *cc*(*i*) be the category that *a*_*i*_ belongs to.

Suppose that, according to N, **T** ^inf^ and **T** ^exam^, *a*_*i*_ ∈ **A** infected *n*_*i*_ other hosts and that there were *m*_*i*_ positive observations of *a*_*i*_. Suppose $H_{i}$ is a phylogenetic tree that describes the part of the outbreak that took place within *a*_*i*_. Because we assume transmission is a complete bottleneck, it is a single tree with a root note *r*. It will have *n*_*i*_ + *m*_*i*_ tips, one for each infection event and each positive observation. If the time of the root *r* is $t_{i}^{\text{root}}$, we know that $t_{i}^{\text{root}}$ is later than $t_{i}^{\text{inf}}$ and we give $H_{i}$ a root branch of length $t_{i}^{\text{root}}-t_{i}^{\text{inf}}$. If we have a $H_{i}$ for each *a*_*i*_, and we know N, we can build a phylogenetic tree for the entire epidemic by, if $a_{j}=N\left(a_{i}\right)$, attaching the root node of $H_{i}$ to the tip of $H_{j}$ that corresponds to the infection of *a*_*i*_ by a branch with length equal to the root branch length of $H_{i}$. If G cannot be built up from $H_{i}\text{s}$ in this way, $p(G|N,T^{\text{inf}},T^{\text{end}},\psi ,T^{\text{exam}})=0$. Otherwise, we calculate it as

$$\begin{array}{c} p\left(G|N,T^{\text{inf}},T^{\text{end}},\psi ,T^{\text{exam}}\right)=\prod_{i\in \{1,\ldots ,N\}}p\left(H_{i}|\psi_{cc(i)}\right). \end{array}$$

In the standard coalescent model [30], the probability density function for the time *t* (in backwards time) of the first coalescence of *K* ≥ 2 lineages after *t*_0_ where the demographic function is *N*_**c**_ is given by

$$\begin{array}{ccc} p(t) & = & \frac{K(K-1)}{2N_{c}\left(t\right)}\text{exp}\left(-\int_{t_{0}}^{t}\frac{K(K-1)}{2N_{c}\left(s\right)}ds\right). \end{array}$$

and if we know which two specific lineages coalesced, the first *K*(*K* − 1)/2 cancels. As Didelot et al. [10] note, this is not quite sufficient for our purposes because we have a maximum height for the last coalescence. If this is *t*_max_, the normalised probability distribution for the time of first coalescence is

$$\begin{array}{c} p\left(t|t_{\text{max}}\right)=\left\{\begin{array}{cc} \frac{\frac{K(K-1)}{2N_{c}\left(t\right)}\text{exp}\left(-\int_{t_{0}}^{t}\frac{K(K-1)}{2N_{c}\left(s\right)}ds\right)}{1-\text{exp}\left(-\int_{t_{0}}^{t_{\text{max}}}\frac{K(K-1)}{2N_{c}\left(s\right)}ds\right)} & t_{0}\leq t<t_{\text{max}}\\ 0 & \text{otherwise.} \end{array}\right. \end{array}$$ (1)

This is the probability of an interval in $H_{i}$ ending in a coalescent event. The probability of an interval ending in a transmission or sampling event is the probability that no events occur in the interval, which is one minus the cumulative distribution function *P*(*t*|*t*_*max*_)

$$\begin{array}{cc} 1-P(t|t_{\text{max}}) & =\left\{\begin{array}{cc} 1 & t<t_{0}\\ \frac{\text{exp}\left(-\int_{t_{0}}^{t}\frac{K(K-1)}{2N_{c}\left(s\right)}ds\right)-\text{exp}\left(-\int_{t_{0}}^{t_{\text{max}}}\frac{K(K-1)}{2N_{c}\left(s\right)}ds\right)}{1-\text{exp}\left(-\int_{t_{0}}^{t_{\text{max}}}\frac{K(K-1)}{2N_{c}\left(s\right)}ds\right)} & t_{0}\leq t<t_{\text{max}}\\ 0 & t\geq t_{\text{max}}. \end{array}\right. \end{array}$$ (2)

Note that while with no maximum root height, the formula happens to work for *K* = 1, here it does not as the denominator is 0 for *t*_0_ ≤ *t* < *t*_*max*_, and we instead set the probability of any interval with one lineage to 1. In particular, if *a*_*i*_ has no children then $p(H_{i}|\psi_{cc(i)})=1$.

These formulae can be used to calculate $p(H_{i}|\psi_{cc(i)})$ for every $H_{i}$ in the established way for a tree with temporally offset tips [23]. It is most intuitive to standardise the timescale of each $H_{i}$ such that the effective population size at the point of the infection can be the same across all hosts. As a result, when (and only when) dealing with within-host phylogenies we depart from the convention of making height 0 the time of the last tip, and instead put it at the time of infection (i.e. *t*_max_ = 0), with all later events occurring at negative heights. Appropriate demographic functions should be picked for the *N*_**c**_s; we suggest exponential or logistic growth [30, 31].

We now calculate the product $p\left(N,T^{\text{inf}},T^{\text{end}}|b,\phi ,\rho ,L\right)p\left(\rho \right)$. The first half, $p(N,T^{\text{inf}},T^{\text{end}}|b,\phi ,\rho ,L)$, is the probability that the observed transmission tree and all its timings occurred for a given *b*, *ϕ* and *ρ*. This can be calculated using a procedure similar to that employed by Deardon et al. [21]. If there are, in addition to the *N* infected hosts *a*_1_, …, *a*_*N*_, *N*′ known potential hosts *a*_*N*+1_, …, *a*_*N*+*N*′_ that were never infected, let *o* be a permutation function such that $t_{o(1)}^{\text{inf}},\ldots ,t_{o(N+N^{\prime })}^{\text{inf}}$ is in increasing order of time (breaking ties arbitrarily and remembering that never-infected hosts are given “infection times” after any others).

For the real infection events, there are *N* − 1 inter-infection intervals *I*_2_, …, *I*_*N*_ where each $I_{i}=\left(t_{o(i-1)}^{\text{inf}},t_{o\left(i\right)}^{\text{inf}}\right]$; let $I_{1}=\left(-\infty ,t_{o(1)}^{\text{inf}}\right]$ and $I_{N+1}=\left(t_{o(N)}^{\text{inf}},t_{o(N+1)}^{\text{inf}}\right]$ (the end of the latter interval is the time of infection assigned to all the never-infected susceptibles). Let S(t) be the set of susceptible hosts, I(t) the set of infected hosts, and R(t) the set of noninfectious hosts at time *t*. For any *i* > 1, $a_{o(i)}\in S\left(t_{o(i-1)}^{\text{inf}}\right)$ and $N\left(a_{o(i)}\right)\in I\left(t_{o(i-1)}^{\text{inf}}\right)$. Recall that we assume that the noninfectiousness time of each host is determined upon infection by a draw from a probability distribution with parameters *ρ*. For any *i* ≤ *N*, the event *E*_*i*_ is taken to represent the combined occurance of a) *a*_*o*(*i*)_ being infected by $N(a_{o(i)})$ at the end of *I*_*i*_, b) the time of removal of *a*_*o*(*i*)_ being $t_{o(i)}^{\text{end}}$, and c) no other infection events taking place during *I*_*i*_. The event *E*_*N*+1_ represents no infections occurring amongst any remaining susceptibles during *I*_*N*+1_. We now derive the probability $p(N,T^{\text{inf}},T^{\text{end}}|b,\phi ,\rho ,L)$:

$$\begin{array}{cc} p(N,T^{\text{inf}},T^{\text{end}}|b,\phi ,\rho ,L) & =p(\{E_{i}:1\leq i\leq N+1\}|b,\phi ,\rho ,L)\\ & =\prod_{i=1}^{N+1}p\left(E_{i}|\{E_{j}:j<i\},b,\phi ,\rho ,L\right). \end{array}$$ (3)

The first term *p*(*E*_1_|*b*, *ϕ*, *L*, *ρ*) is the product of $p(t_{o(1)}^{\text{end}}|t_{o(1)}^{\text{inf}},\rho )$, and the probability that *a*_*o*(1)_ was infected at time $t_{o(1)}^{\text{inf}}$ and was the first in the epidemic. The latter should be defined by a prior; call this $p(a_{\text{index}},t_{\text{index}}^{\text{inf}})$. For *E*_*i*_ with 2 ≤ *i* ≤ *N*:

- Let *X*_*i*_ be the probability $N(a_{o(i)})$ infected *a*_*o*(*i*)_ at $t_{o(i)}^{\text{inf}}$, but not before that during *I*_*i*_:
  $$\begin{array}{c} X_{i}=bF\left(a_{o(i)},N\left(a_{o(i)}\right)\right)\times \text{exp}\left(-bF\left(a_{o(i)},N\left(a_{o(i)}\right)\right)\left(t_{o(i)}^{\text{inf}}-t_{o(i-1)}^{\text{inf}}\right)\right). \end{array}$$
- Let *Y*_*i*_ be the probability that no host in $I(t_{o(i-1)}^{\text{inf}})$ other than $N(a_{o(i)})$ infected *a*_*o*(*i*)_ before $t_{o(i)}^{\text{inf}}$ in *I*_*i*_. Noting that the upper bound on the time that such an *a*_*j*_ could have infected *a*_*o*(*i*)_ before $t_{o(i)}^{\text{inf}}$ is either $t_{o(i)}^{\text{inf}}$ itself if *a*_*j*_ was still infectious at that point or $t_{j}^{\text{end}}$ if it was not, this is given by
  $$\begin{array}{c} Y_{i}=\prod_{\begin{array}{l} a_{j}\in I\left(t_{o\left(i-1\right)}^{\text{inf}}\right)\\ a_{j}\neq N\left(a_{o\left(i\right)}\right) \end{array}}\text{exp}\left(-bF\left(a_{o(i)},a_{j}\right)(\text{min}\{t_{o(i)}^{\text{inf}},t_{j}^{\text{end}}\}-t_{o(i-1)}^{\text{inf}})\right). \end{array}$$
- Let *Z*_*i*_ be the probability that no host in $I(t_{o(i-1)}^{\text{inf}})$ infected any host other than *a*_*o*(*i*)_ in $S(t_{o(i-1)}^{\text{inf}})$ (a set that always includes all the never-infected susceptibles) during *I*_*i*_. Again, the upper bound on the time at which an *a*_*j*_ could infect a third host *a*_*k*_ before $t_{o(i)}^{\text{inf}}$ is $\text{min}\{t_{o(i)}^{\text{inf}},t_{j}^{\text{end}}\}$.
  $$\begin{array}{c} Z_{i}=\prod_{a_{j}\in I\left(t_{o(i-1)}^{\text{inf}}\right)}\prod_{\begin{array}{l} a_{k}\in S\left(t_{o\left(i-1\right)}^{\text{inf}}\right)\\ k\neq o\left(i\right) \end{array}}\text{exp}\left(-bF\left(a_{j},a_{k}\right)(\text{min}\{t_{o(i)}^{\text{inf}},t_{j}^{\text{end}}\}-t_{o(i-1)}^{\text{inf}})\right). \end{array}$$

Then $p\left(E_{i}|\{E_{j}:j<i\},b,\phi ,\rho ,L\right)=X_{i}Y_{i}Z_{i}p\left(t_{o(i)}^{\text{end}}|t_{o(i)}^{\text{inf}},\rho \right)$.

Finally, consider *E*_*N*+1_. After $t_{o(N)}^{\text{inf}}$, the only remaining susceptibles were never infected, and their assignment of $t_{o(N+1)}^{\text{inf}}$ as an infection date is a consequence of this. If *W* = *p*(*E*_*N*+1_|{*E*_*i*_: *i* < *N*+1}, *b*, *ϕ*, *ρ*, *L*), then it is just the probability that no never-infected susceptible is infected after $t_{o(N)}^{\text{inf}}$ (the probability that these were not infected earlier is handled in the construction of *Z*_*i*_ above) and this is given by

$$\begin{array}{c} W=\prod_{a_{j}\in I\left(t_{o(N)}^{\text{inf}}\right)}\prod_{a_{k}\in S\left(t_{o(N)}^{\text{inf}}\right)}\text{exp}\left(-bF\left(a_{j},a_{k}\right)(t_{j}^{\text{end}}-t_{o(N)}^{\text{inf}})\right). \end{array}$$

The time period after $t_{o(N+1)}^{\text{inf}}$ need not be considered. If the epidemic ended with the host *a*_*j*_ ceasing to be infectious at $t_{j}^{\text{end}}<t_{o(N+1)}^{\text{inf}}$, the infectious pressure applied to all never-infected susceptibles after $t_{j}^{\text{end}}$ will be zero permanently and hence the probability that they were not infected after $t_{j}^{\text{end}}$, if they were not infected before it, is 1. All other hosts were removed by this time and cannot be reinfected. If, on the other hand, any hosts remain infectious at the time of analysis, the probability of no infection for any member of the never-infected set must be calculated up to that time, which is, by construction, $t_{o(N+1)}^{\text{inf}}$. This method is not designed to infer future events, so the timeline is truncated at this point.

We multiply the conditional probabilities of each *E*_*i*_ together to form Eq (3) and we have

$$\begin{array}{ccc} p\left(N,T^{\text{inf}},T^{\text{end}}|b,\phi ,\rho ,L\right)p\left(\rho \right) & = & p\left(a_{\text{index}},t_{\text{index}}^{\text{inf}}\right)\left(W\prod_{i=2}^{N}X_{i}Y_{i}Z_{i}\right)\\ & & \times \left(\prod_{i=1}^{N}p\left(t_{o(i)}^{\text{end}}|t_{o(i)}^{\text{inf}},\rho \right)\right)p\left(\rho \right). \end{array}$$

*W* and each product *X*_*i*_ *Y*_*i*_ *Z*_*i*_ can be calculated individually. A term $p(t_{i}^{\text{end}}|t_{i}^{\text{inf}},\rho )$ can be seen as the probability that the infectious period of *a*_*i*_ has length $t_{i}^{\text{end}}-t_{i}^{\text{inf}}$ given *ρ*. Writing $l_{i}^{\text{inf}}=t_{i}^{\text{end}}-t_{i}^{\text{inf}}$ and assuming infectious periods are independent of each other, then

$$\begin{array}{cc} \left(\prod_{i=1}^{N}p\left(t_{o(i)}^{\text{end}}|t_{o(i)}^{\text{inf}},\rho \right)\right)p\left(\rho \right) & =p\left(l_{1}^{\text{inf}},\ldots ,l_{N}^{\text{inf}}|\rho \right)p\left(\rho \right). \end{array}$$

There are two ways to handle this term. The simpler is to treat *ρ* as known, in which case *p*(*ρ*) = 1 and what we are effectively doing is placing a prior distribution on the length of each infectious period. No assumption is made that each $l_{i}^{\text{inf}}$ is drawn from the same distribution; indeed every single infectious period can be treated as coming from a different distribution. Previous work on foot-and-mouth disease virus [5, 7] has used clinical data to estimate times of infection, and if this kind of information is available, it can be used to determine an individual prior for each *l*_*i*_. This is also the preferable approach if infections are ongoing at the time of sampling. If we cannot use information of this type, a similar approach can be taken to that in the coalescent calculations above, assigning each host *a*_*i*_ to an infectious period category *ic*(*a*_*i*_). This again allows us to accommodate known heterogeneity; for example in an agricultural outbreak it is likely that infectious periods decrease as time goes by and control measures are brought to bear.

It may be, however, that we want to estimate the distribution of infectious periods from the genetic data. Suppose hosts in category *A* have infectious periods distributed according to a distribution *D*_*A*_ with unknown parameters *ρ*_*A*_. In this case *p*(*ρ*_*A*_) would be determined by hyperpriors. We suggest that, as an alternative to using MCMC to estimate both the parameters of *D*_*A*_ and a set of draws from it, the actual values of *ρ*_*A*_ be integrated out by appealing to a conjugate prior distribution for *D*_*A*_ and calculating the marginal likelihood of the set {*l*_*i*_: *ic*(*a*_*i*_) ∈ *A*} given the hyperpriors. Candidates for *D*_*A*_ are then those continuous distributions for which this marginal likelihood is analytically tractable. Examples are normal, lognormal, exponential, and gamma if the shape parameter is known. Although it it not absolutely ideal as infectious periods are non-negative parameters, we suggest the normal distribution as the prior for the reason that its mean and variance are independent.

Finally, all that remains is to place prior distributions on the parameters making up *b*, *ϕ*, *ψ*, and *ω*.

### An alternative approach

If it is reasonable to assume that all hosts cease to be infectious upon examination (and all examinations of each host take place simultaneously), then **T** ^end^ and **T** ^exam^ coincide (meaning that we no longer must assume examination does not disturb the transmission process) and we can replace the latter with a term **N** ^exam^ which simply counts the number of sequences taken from each host. Negative examinations no longer need to be considered, and *D* is simply sequence data. A tree with a number of tips other than *M* has zero prior probability as well as zero likelihood because we condition on **N** ^exam^. The calculations are identical except that the initial check for consistency of the number of examinations with **T** ^inf^ and **T** ^end^ can be skipped.

### Latent periods

The above formulation has taken the course of infection to follow a SIR process; hosts are assumed to be infectious as soon as they are infected. It is straightforward to replace this with a SEIR process instead. While it is possible to treat latent periods as draws from a probability distribution in the same way as described for infectious periods in the previous section, in simulations this resulted in poor mixing of the MCMC chain if an strongly informative hyperprior on the parameters of this distribution was used, and poor estimation of their values if the hyperprior was weaker. Instead, we again subdivide the set of hosts into one or more discrete categories and assign a single value to the latent period for all hosts in each category, so that the latent period of host *a*_*i*_ is *lc*(*a*_*i*_). Let the complete set of latent periods be *λ*. Let **T** ^*trans*^ be the set of infectiousness times of each host; then if $t_{i}^{\text{trans}}\in T^{\text{trans}}$ is the infectiousness time of *a*_*i*_, $t_{i}^{\text{trans}}=t_{i}^{\text{inf}}+lc\left(a_{i}\right)$. We assume that hosts are infectious by the time they cease to be infected, and that examinations of infected but noninfectious hosts are positive. The phylogeny G is assumed to be conditionally independent of **T** ^trans^ given **T** ^inf^ and **T** ^end^.

The new decomposition is

$$\begin{array}{ccc} p(G,N,T^{\text{inf}},T^{\text{end}},T^{\text{trans}},\phi ,\psi ,\rho ,\lambda ,\omega |T^{\text{exam}},L) & = & p(G|N,T^{\text{inf}},T^{\text{end}},\psi ,T^{\text{exam}})\\ & & \times p(N,T^{\text{inf}},T^{\text{trans}},T^{\text{end}}|b,\phi ,\rho ,\lambda ,L)\\ & & \times p(b)p(\phi )p(\psi )p(\rho )p(\lambda )p(\omega ). \end{array}$$

Aside from the prior on *λ*, only the second term in this product is different, and it is not a major modification to the SIR version to calculate it; see S2 Text.

### Simulations

Epidemics and sequences were simulated using examples of the three models described above. The epidemic simulations were intended to represented a situation analogous to an agricultural outbreak, with the hosts as farms. The units of time were intended to represent days. In each replicate of the simulation, **A** consisted of 50 potential hosts arranged spatially on a regular 5 × 5 grid contained in the unit square, such that every grid point contained two whose distance from each other was zero. A single host was chosen at random to be infected first at time 0. The infection of each followed a SEIR process: upon infection, a host *a*_*i*_ was latently infected for a time *P* ^lat^ which was identical across all hosts and subsequently infectious for a period $p_{i}^{\text{inf}}$ drawn from a normal distribution (negative draws were discarded, but the distribution used was such that the probability of these occurring was negligible). Let **P** ^inf^ be the set of all the infectious periods.

*F* was an exponential spatial transmission kernel function: the time for an newly infectious *a*_*i*_ to infect a susceptible *a*_*j*_ was drawn from an exponential distribution with mean *be* ^− *αd*(*a*_*i*_, *a*_*j*_)^ where *d*(*a*_*i*_, *a*_*j*_) is the Euclidean distance between the locations of *a*_*i*_ and *a*_*j*_. The process was run until no infections remained. A single positive examination was simulated at the point of noninfectiousness of each host. As no infections persisted following the acquisition of a sequence, we are in the special case outlined under “an alternative approach” above and so there is no need to consider the possibility of negative examinations in the analysis. Only simulations in which at least 45 of the 50 susceptibles (i.e. *N* ≥ 45) were eventually infected were kept.

Once the epidemic simulation was completed, the transmission tree was transformed into a phylogenetic tree by simulating a within-host phylogeny under a coalescent process. Variation in the product of effective population size and generation time of the agents within each host was identical and obeyed a logistic growth function *N*_*e*_(*t*):

$$\begin{array}{c} N_{e}\left(t\right)=\frac{N_{0}\left(1+e^{-rT_{50}}\right)}{1+e^{-r(T_{50}-t)}}. \end{array}$$

where the timescale is in negative time and distinct for each host and *t* = 0 is the point of infection. *N*_0_ represents the effective population size at *t* = 0, *r* the growth rate during the exponential growth phase of the logistic function, and *T*_50_ the time such that *N*_*e*_(*T*_50_) is half the value of the limit of *N*_*e*_(*t*) as it approaches −∞. We conditioned the simulation on all lineages coalescing before *t* = 0. The complete set of such phylogenies was then joined up to produce a single phylogeny for the entire simulated epidemic.

This full phylogeny was then used to generate simulated sequences using the program πBUSS [32]. Sequences consisted of 14,000 base pairs (roughly equivalent to a full influenza A genome). A strict molecular clock model with no rate variation between sites and equal nucleotide frequencies was used. Two sets of sequences were generated. The first used an unrealistically fast molecular clock with a rate of 5 ⋅ 10^−4^ substitutions per site per day (0.183 per site per year) while the second had a rate of 1 ⋅ 10^−5^ per site per day (3.65 ⋅ 10^−3^ per site per year). The slower rate was intended to be be similar to the genuine substitution rate for influenza A. Both used the HKY substitution model [26] with a *κ* value of 2.718. Table 2 gives the parameter values actually used in the simulations.

**Table 2:** Explanation of the mathematical symbols used in the simulation model, and prior distributions for their values used in analysis of the simulated datasets. Mathematical symbols are given where they appear in the text.

Symbol	Meaning	Actual Value	Prior distribution
*a*_index_	Identity of index host	variable	U{1,N}
$t_{\text{index}}^{\text{inf}}$	Infection time of index host	0	N(0,2)
*α*	Transmission kernel dispersion parameter	10	exp(1)
*b*	Unmodified infection rate	0.1/day	exp(0.5)
*r*	Within-host logistic growth rate	1.5/day	None^1^
*N*_0_	*N*_*e*_ at time of infection	0.1	None^2^
*T*_50_	Time before time of infection at which *N*_*e*_ achieves half its limit	-4 days	Gamma(10, 2)
*S*	Ratio of lim_*t* → −∞_ *N*_*e*_(*t*) to *N*_0_	55.6	lnN(4,0.5)
*P* ^lat^	Latent period	2 days	Gamma(200, 100)
*μ*_inf_, *τ*_inf_	Mean and precision of normal distribution of infectious periods	10 days, 1 days^−2^	NormalGamma(10, 0.01, 1, 1)^3^
	Molecular clock rate, fast clock datasets	5 ⋅ 10^−4^/site/day	exp(0.1)
	Molecular clock rate, slow clock datasets and prior analysis	1 ⋅ 10^−5^/site/day	None^4^
*κ*	HKY model transition/transversion ratio	2.718	lnN(1,0.64)

^1^ The prior probability of *r* is implicitly specified by the priors on *T*_50_ and *S*

^2^ *N*_0_ was fixed to its correct value of 0.1 in the analysis

^3^ The slow clock analysis was also repeated with NormalGamma(10, 100, 1, 1) instead

^4^ In the analyses of the slow clock datasets and the analyses sampling from the prior distribution only, *R* was fixed to its correct value of 1 ⋅ 10^−5^/site/day

Sequence datasets from a total of 25 simulation replicates were used for analysis. We used the within-host coalescent (WHC) method outlined in the previous sections, implemented in BEAST, to reconstruct the full phylogeny and transmission tree for each replicate, and estimate the parameters of the model that generated them. We also performed the same analysis using a blank alignment, sampling from the prior distribution only. Uninfected susceptibles were included in the analysis. For comparison, we also reconstructed the phylogeny only using a GMRF Bayesian skyride [33] tree prior. Table 2 also details the prior distributions used on all parameters. In this paper we concentrate primarily on the between-host model, so the chosen priors on the within-host parameters were somewhat informative about their known values. In the prior, the identity of the index host and its time of infection were taken to be independent, so $p\left(a_{\text{index}},t_{\text{index}}^{\text{inf}}\right)=p\left(a_{\text{index}}\right)p\left(t_{\text{index}}^{\text{inf}}\right)$. A couple of points warrant further explanation.

Firstly, in the reconstruction we assumed that all infectious periods are drawn from an unknown normal distribution with mean *μ*_inf_ and precision *τ*_inf_ and placed a conjugate NormalGamma(*μ*_0_, *κ*_0_, *α*_0_, *β*_0_) hyperprior on *μ*_inf_ and *τ*_inf_. The meaning of this is that *τ*_inf_ is gamma distributed with shape *α*_0_ and rate *β*_0_, and for a known value of *τ*_inf_, *μ*_inf_ is normally distributed with mean *μ*_0_ and precision *κ*_0_ *τ*_inf_. Initial analyses of both datasets had *μ*_0_ = 10, *κ*_0_ = 0.01, *α*_0_ = 1, *β*_0_ = 1. While this value of *μ*_0_ is equal to the actual mean of the distribution used to generate the simulations, the low *κ*_0_ actually means that this hyperprior is only very weakly informative about *μ*_inf_. As it proved that for datasets generated with the slower clock this resulted in a systematic underestimation of the length of infectious periods (see Results), the analysis was repeated with *κ*_0_ = 100, a modification which makes the hyperprior much more informative about *μ*_inf_.

Secondly, very large values of the probability expressions Eqs (1) and (2) can be obtained when their denominators are very small. This occurs when the coalescence of all lineages before the point of infection (in backwards time) is actually highly unlikely given the parameters of the within-host model (because the denominators are the probability of coalescence of all lineages before infection), contrary to the assumption that transmission is a complete bottleneck. There is therefore a mismatch between this bottleneck assumption and some values of the parameters making up *ψ*, and this must be remedied by appropriate prior distributions for the latter; uninformative priors are completely inappropriate. The nature of the mathematics of the coalescent process used here is such that no values will literally make the bottleneck complete, so we instead ensure that it is not unreasonably wide. The ratio *S* of the final asymptotic value of *N*(*t*) to *N*_0_, its value at the point of infection, in our logistic model is

$$\begin{array}{ccc} S & = & \frac{\lim_{t\to -\infty }N\left(t\right)}{N_{0}}\\ & = & 1+e^{-rT_{50}}. \end{array}$$

The concerning situation is where *S* is small. If *T*_50_ is positive then *S* cannot be greater than 2, so we assume it is negative. We then place a lognormal prior on *S*. This prior, combined with one on either *T*_50_ or *r*, specifies the prior probability of *r* so we give the latter no explicit distribution. We also fixed *N*_0_ to its correct value in all simulations.

In analysing the sequence datasets generated by the slower molecular clock, the amount of genetic variation accumulated over the timescale of each epidemic was found to be insufficient, for some simulations, to provide good estimates of the clock rate. As a result, this parameter was also fixed to its correct value. The same was done for the prior analysis. All MCMC chains were run for sufficiently long to give effective sample sizes of at least 200 for all numerical model parameters.

Accuracy of the reconstructed phylogenetic tree topology was assessed by counting, for each tree in the posterior sample, the number of subtree prune and regraft (SPR) moves required to take it to the correct phylogeny and taking the posterior median value of this count; we used the program rSPR [34] to determine this. In addition, to investigate the extent to which the imposition of a transmission model constrains the space of plausible phylogenies, we calculated the number of unique clades in the 50% credible set of phylogenies for the WHC and skyride analyses of each slow and fast clock dataset.

We used two methods to assess procedures by which transmission tree might be reconstructed in practice. Firstly, the posterior set of trees was summarised in a single maximum parent credibility (MPC) transmission tree, analogous to the maximum clade credibility (MCC) tree for phylogenies. The posterior distribution of parents for each host in the epidemic was calculated for each host in turn, and the parent credibility of each tree in the sample was calculated as the product of the posterior probabilities of each link in the chain. The MPC tree is the tree in the sample that maximises this product. This was compared to the correct transmission tree, and the proportion of parents that were correctly identified calculated.

As an alternative approach we identified, for each host, the infector with the highest posterior probability, regardless of whether the result of doing this for every host actually constituted a proper transmission tree that was connected with no cycles. We calculated the proportion of parents that would be correctly identified by doing this, firstly if the actual value of the posterior probability was not considered, and subsequently for different values of a threshold probability below which inference of parental relationships would not be made.

### Analysis of sequences from the 2003 H7N7 avian influenza outbreak in the Netherlands

We used the WHC method to reanalyse the data from the Dutch H7N7 avian influenza outbreak of 2003. The outbreak has been the subject of many previous papers [35–37], including several that incorporated genetic data [6, 18, 38–40].

Epidemiological data from the Dutch epidemic consisted of cull dates for all 241 farms, and the matrix of spatial distances between them, rounded to the nearest kilometer. (We did not have access to their precise spatial locations.) The GISAID database [41] contains sequences for isolates taken from 229 of the farms (95.0%); this consists of the HA, NA and PB2 segments in 226 cases, the HA and PB2 in 2, and the HA and NA in 1. The dates upon which these samples were taken were also available. In the absence of any other information, we assumed that a single examination of each farm took place at this time.

The HA, NA and PB2 sequences were each aligned using the MUSCLE algorithm [42]; segments which were missing were given noninformative sequences consisting entirely of the code “N”. This included entirely noninformative sequences for the twelve farms for which we had no genetic data at all; the examination date of these was set to the cull date of the farm, a time at which it was certainly possible to acquire a sequence. The three segments were then concatenated to produce a single alignment. The 143rd codon position of the HA segment, which has been observed to cause discrepancies between reconstructed phylogenies for each segment probably as the result of convergent evolution [18], was removed. As we lacked data on the location of uninfected farms in the country, we did not include uninfected premises in the analysis, and as a result we were estimating *b*′ and *ϕ*′ (see Bayesian Decomposition).

The parameters of this analysis, and the prior distributions used for them, are summarised in Table 3. We used the SRD06 nucleotide substitution model [43] and an uncorrelated lognormal relaxed molecular clock [29]; the mean clock rate was not fixed *a priori*. The type of spatial transmission kernel function used here was the same as that used by Boender et al. [37] in their analysis of the same epidemic, determined by a logistic expression

$$\begin{array}{c} F\left(a_{i},a_{j}\right)=\frac{1}{1+{\left(\frac{d(a_{i},a_{j})}{\alpha_{2}^{\prime }}\right)}^{\alpha_{1}^{\prime }}}. \end{array}$$

where *d*(*a*_*i*_, *a*_*j*_) is the distance between the farms *a*_*i*_ and *a*_*j*_. As before, the latent period of the disease was assumed to be constant, and we placed a strong prior with a mean of two days on its length. We also followed Boender et al. in assuming that the distribution of farm infectious periods prior to the discovery of the epidemic and the implementation of control measures was distinct from that afterwards, and grouped the set of farms into “high-risk” and “low-risk” categories accordingly. The first five detected cases (F1–F5) were in the high-risk category. The hyperpriors on the distribution of infectious periods in both categories and the prior distribution for the time of the initial infection were informed by estimates from Boender et al. and Stegeman et al. [36]. The prior distribution for the identity of the index farm was such that each high-risk farm was given ten times the weight of each low-risk farm.

**Table 3:** Parameters used in the H7N7 analysis, and prior distributions for their values.

Parameter	Symbol	Prior distribution
Identity of index host	*a*_index_	$p\left(a_{i}=a_{\text{index}}\right)=\{\begin{array}{cc} 0.035 & a_{i}\;\text{high-risk}\\ 0.0035 & a_{i}\;\text{low-risk} \end{array}$
Infection time of index host	$t_{\text{index}}^{\text{inf}}$	N(E,2) ^1^
Transmission kernel dispersion parameters^2^	$\alpha_{1}^{\prime },\alpha_{2}^{\prime }$	U(0,∞)
Unmodified transmission rate^2^	*b*′	U(0,∞)
Within-farm logistic growth rate	*r*	None^3^
Product of effective population size and pathogen generation time at point of infection	*N*_0_	Gamma(20, 4)
Time before infection time at which *N*_*e*_ achieves half its final asymptotic value	*T*_50_	None^4^
Ratio of lim_*t* → −∞_ *N*_*e*_(*t*) *N*_0_	*S*	lnN(4,0.5)
Latent period	*P* ^lat^	Gamma(200, 100)
Mean and precision of normal distribution of infectious periods, high-risk period		NormalGamma(7.3, 169.0, 1, 3.8)
Mean and precision of normal distribution of infectious periods, low-risk period		NormalGamma(13.8, 2.64, 1, 3.8)
Mean molecular clock rate (real space)		U(0,∞)
Standard deviation parameter of relaxed molecular clock (log space)		Exp(0.33)
Transition/transversion ratio		lnN(1,0.64)
Shape parameter of gamma distribution for between-site rate variation		Exp(0.5)
Nucleotide frequencies		U(0,1)
Relative clock rates for nucleotide positions 1+2 and 3		U(0,∞)

^1^ *E* corresponds to 17 February 2003, an estimate for the time of the index infection taken from previous literature [36].

^2^ These parameters is not the true values that that would be estimated in the presence of data on uninfected susceptibles; see the text for details.

^3^ The prior probability of *T*_50_ is implicitly specified by the priors on *r* and *S*

^4^ *N*_0_ was fixed to 3.37 in the analysis

We chose to regard the agent population as being made up of infected birds. As in the simulations, we assumed that the product of the effective size and the generation time of this population within each farm underwent logistic growth, and that the same growth function was shared by all farms. Also as in the simulations, we did not estimate *N*_0_ and instead assumed that the effective population size at the point of infection was 1, and that the generation time (the serial interval of the infection) was 3.37 days, a number derived from White and Pagano [44].

Multiple MCMC runs were performed, and the results combined using the LogCombiner utility in order to achieve ESS values over 200 for the posterior and prior probabilities, the likelihood, and all parameters listed in Table 3. The MPC transmission tree was visualised with Cytoscape 3.2 [45].

## Results

### Simulations

**Fig 3:**
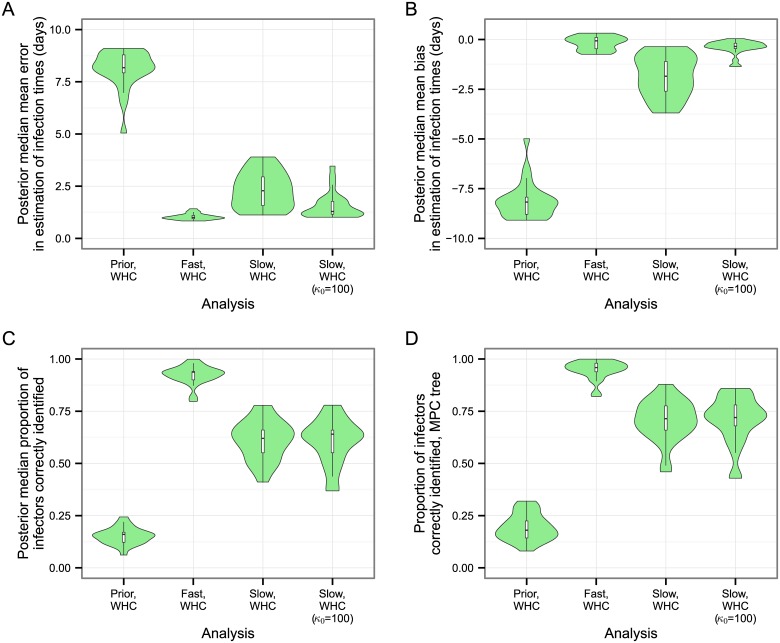
Accuracy of the reconstruction of the transmission tree. Each violin plot represents the density of a statistic calculated from the results of separate analyses of 25 simulated datasets; the clock model used to generate the dataset and the analysis method are indicated on the *y*-axis. (A) posterior median of mean bias in estimation of infection dates. (B) posterior median of mean error in estimation of infection dates. (C) Posterior median proportion of hosts whose infector is correctly identified. (D) Proportion of hosts whose infector is correctly identified in the maximum parent credibility (MPC) transmission tree.

The simulation needed to be run only 26 times in order to obtain 25 instances in which at least 45 hosts were infected, suggesting that there was little bias involved in discarding those that failed to meet this threshold. Fig 3 summarises the accuracy of the reconstruction of the transmission tree and the estimates of infection times for each host. For the latter, we saw low bias and error when the molecular clock rate was fast. However, the use of realistic sequences led to a systematic tendency to underestimate times from infectiousness to removal when the mean parameter of the probability distribution from which infectious periods are drawn was not given a strongly informative prior. It is clear from the results of the prior analysis that the effective prior distribution favours short infected periods. Re-running the analysis with an informative prior on *μ*_inf_ (by setting *κ*_0_ = 100, see Methods) greatly reduced this effect, but did not entirely eliminate it.

The transmission tree was very well reconstructed when the clock was fast, with the posterior median proportion of parents being correctly identified, across the 25 simulations, having a median of 0.94 (range 0.8–1). For the slower clock this was considerably reduced, with a median of 0.64 (0.46–0.78). Increasing *κ*_0_ had no noticeable effect on this (median 0.62, range 0.37–0.78). As expected, reconstruction of the transmission tree when MCMC samples were taken from the prior distribution only was extremely poor. The MPC transmission tree’s median proportion of correctly identified parents was 0.96 (0.82, 1.00) for the fast clock dataset, 0.71 (0.46, 0.88) for initial slow clock dataset, and 0.72 (0.43, 0.86) for the slow clock dataset with *κ*_0_ = 100.

Table 4 summarises the accuracy of the procedure of picking the infector with the highest posterior probability, for no probability threshold and thresholds of 0.5, 0.8, and 0.9. It can be seen that for a threshold of 0.8, inferences are highly accurate even for the slow clock dataset and that the use of a value of this size leaves up to two-thirds of hosts with an inferred infector.

**Table 4:** Percentage of hosts with parents correctly identified by picking the infector host with the highest posterior probability for different thresholds, and percentage of hosts whose parents are inferred in this way for each threshold. Numbers are median and range across the 25 simulations.

Analysis	Statistic	Threshold			
		None	0.5	0.8	0.9
Prior	% Parents correctly identified	22 (10, 28.5)	40 (25, 75)	66.7 (0, 100)	75 (0, 100)
	% Parents inferred	100	21.3 (10, 36)	8.0 (2.0, 16.7)	6.0 (2.0, 14.6)
Fast clock	% Parents correctly identified	95.9 (82.0, 100)	95.9 (82, 100)	97.8 (85.7, 100)	100 (88.9, 100)
	% Parents inferred	100	100 (97.8, 100)	91.7 (79.6, 100)	84.0 (68.0, 100)
Slow clock	% Parents correctly identified	72.0 (54.2, 88)	84.2 (69, 100)	94.1 (85.7, 100)	100 (86.4, 100)
	% Parents inferred	100	76 (42.9, 94)	42.9 (16.3, 64)	28 (4.1, 50)
Slow clock, *κ*_0_ = 100	% Parents correctly identified	70.2 (52.1, 86)	85.3 (67.6, 95.7)	96 (85.2, 100)	100 (85.7, 100)
		% Parents inferred	100	75.5 (36.7, 92)	41.7 (4.1, 64)	28.3 (2, 54)

**Fig 4:**
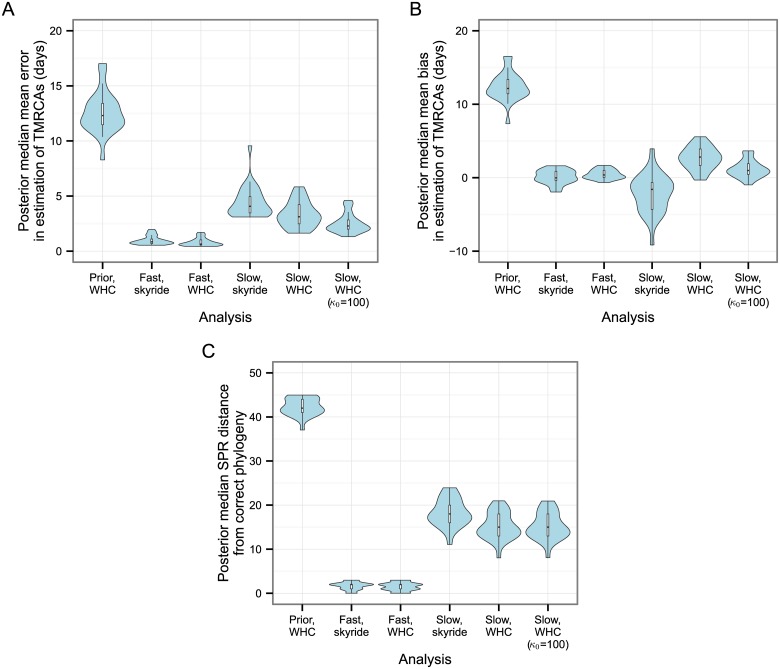
Accuracy of the reconstruction of the phylogeny. Each violin plot represents the density of a statistic calculated from the results of separate analyses of 25 simulated datasets; the clock model used to generate the dataset and the analysis method are indicated on the *y*-axis. (A) posterior median of mean bias in estimation of all pairwise TMRCAs. (B) posterior median of mean error in estimation of all pairwise TMRCAs. (C) Posterior median SPR distance from the true phylogeny.

For the fast clock sequences, the phylogeny was sufficiently well resolved by the genetic data that both methods, the established skyride coalescent tree prior and the WHC introduced in this paper, performed similarly in reconstructing it, but WHC performed better when the molecular clock rate was more realistic (Fig 4). Error and bias in the estimates of the TMRCA of each pair of sequences was notably reduced for WHC. Using an informative prior on *μ*_inf_ made estimates better still. The reconstruction of the topological structure of the phylogeny was also improved, with the number of SPR moves needed to take a sampled tree from the MCMC chain to the true phylogeny being consistently smaller for WHC, where the median (across the 25 simulations) posterior median number of required SPR moves was 15 (range 8–21), compared to the skyride analysis, where it was was 18 (range 11–24). The informative prior on *μ*_inf_ made no noticeable difference in this case. In the slow clock analyses, the number of unique clades in the 50% credible set of phylogenies was always larger when the skyride was used than when the WHC was, sometimes by a factor of as much as 2; the median ratio of the former to the latter was 1.87 (range 1.19–2.78). For the fast clock datasets, however, the numbers from each method were extremely similar, indicating the extent to which the phylogeny could be resolved by the genetic data alone.

Table 5 summarises the posterior parameter estimates and their accuracy. Figures given are the medians across the 25 simulations. The tendency of WHC to substantially underestimate infectious periods unless an informative prior is used on the mean of their distribution is also clear here; latent periods were also slightly underestimated although the true values were always well within the 95% highest posterior density (HPD) interval. It is also noticeable that the parameters *r* and *T*_50_ of the logistic growth function describing within-host effective population size are not well estimated for the slow clock dataset, with very wide HPD intervals and also a great bias towards underestimating the value of the latter, to the extent that the 95% HPD was frequently inaccurate. The informative prior on *μ*_inf_ improved matters somewhat, at least ensuring that the true *T*_50_ usually lay within the HPD interval. On the other hand, the ratio *S* was recovered with much more precision and much less error and bias. These within-host parameters were in fact rather better estimated when sampling was from the prior only, but this is presumably because the prior distributions on them were chosen with knowledge of their true values. To investigate this further, we re-ran the analyses on the fast and slow clock datasets (with *κ*_0_ = 0.01), fixing the parameters of the within-host model to their true values, and once again determined the accuracy of the reconstruction of the transmission tree. The results of this can be seen in S2 Fig. For the slow clock dataset, this improved the reconstruction somewhat, although the effect was not dramatic.

**Table 5:** Estimates of simulation parameters from the various analyses. The median values, across the 25 simulations, of the posterior median, relative error in the posterior median, relative bias in the posterior median and relative width of the 95% HPD interval of each parameter are given, along with the number out of 25 simulations that the correct value was contained within the 95% HPD interval. Where estimates are not given for a particular analysis, this parameter was either fixed to its correct value or, in the case of WHC-related parameters in skyride analyses, not part of the analysis. Mathematical symbols are given where they are referred to in the text.

Symbol	Meaning	Dataset	Model	True value	Median	Error	Bias	95% HPD width	HPD accuracy
	Molecular clock rate^1^	Fast	Skyride	5 ⋅ 10^−4^	5.11 ⋅ 10^−4^	2.68 ⋅ 10^−2^	2.13 ⋅ 10^−2^	0.125	24
		Fast	WHC	5 ⋅ 10^−4^	5.1 ⋅ 10^−4^	2.32 ⋅ 10^−2^	1.91 ⋅ 10^−2^	0.112	23
*κ*	Transition/transversion ratio	Prior	WHC	2.72	2.59	4.67 ⋅ 10^−2^	−4.67 ⋅ 10^−2^	7.41	25
		Fast	Skyride	2.72	2.72	2.12 ⋅ 10^−2^	−1.09 ⋅ 10^−3^	0.113	23
		Fast	WHC	2.72	2.72	2.16 ⋅ 10^−2^	6.58 ⋅ 10^−4^	0.114	23
		Slow	Skyride	2.72	2.52	0.132	−7.13 ⋅ 10^−2^	0.745	24
		Slow	WHC	2.72	2.51	0.13	−7.53 ⋅ 10^−2^	0.77	24
		Slow	WHC (*κ*_0_ = 10)	2.72	2.53	0.129	−7.04 ⋅ 10^−2^	0.763	24
$\bar{P^{\text{inf}}}$	Mean infectious period^2^	Prior	WHC	10	1.99	0.802	−0.802	0.141	0
		Fast	WHC	10	9.85	2.18 ⋅ 10^−2^	−7.88 ⋅ 10^−3^	0.161	25
		Slow	WHC	10	8.26	0.177	−0.177	0.309	13
		Slow	WHC (*κ*_0_ = 10)	10	9.87	1.9 ⋅ 10^−2^	−1.8 ⋅ 10^−2^	0.134	24
*σ*(**P** ^inf^)	Standard deviation of infectious periods^2^	Prior	WHC	1	0.944	0.216	−0.107	0.593	18
		Fast	WHC	1	1.06	0.12	3.13 ⋅ 10^−2^	0.774	25
		Slow	WHC	1	1.63	0.724	0.724	1.99	22
		Slow	WHC (*κ*_0_ = 10)	1	1.39	0.373	0.373	2.29	20
*P* ^lat^	Latent period	Prior	WHC	2	1.78	0.112	−0.112	0.257	17
		Fast	WHC	2	1.98	1.12 ⋅ 10^−2^	−9.13 ⋅ 10^−3^	0.264	25
		Slow	WHC	2	1.93	3.75 ⋅ 10^−2^	−3.75 ⋅ 10^−2^	0.268	25
		Slow	WHC (*κ*_0_ = 10)	2	1.86	6.88 ⋅ 10^−2^	−6.88 ⋅ 10^−2^	0.254	25
*α*	Transmission kernel dispersion parameter	Prior	WHC	7	4.03	0.423	−0.425	0.682	9
		Fast	WHC	7	6.72	6.43 ⋅ 10^−2^	−4.04 ⋅ 10^−2^	0.469	23
		Slow	WHC	7	6.81	6.87 ⋅ 10^−2^	−2.69 ⋅ 10^−2^	0.53	24
		Slow	WHC (*κ*_0_ = 10)	7	6.90	5.67 ⋅ 10^−2^	−1.41 ⋅ 10^−2^	0.538	24
*b*	Unmodified transmission rate	Prior	WHC	0.1	0.143	0.462	0.43	2.49	25
		Fast	WHC	0.1	9.88 ⋅ 10^−2^	0.186	−1.24 ⋅ 10^−2^	1.03	24
		Slow	WHC	0.1	0.111	0.182	0.105	1.43	25
		Slow	WHC (*κ*_0_ = 10)	0.1	0.103	0.186	3.1 ⋅ 10^−2^	1.31	25
*r*	Within-host logistic growth rate	Prior	WHC	1	0.75	0.25	−0.25	2.84	25
		Fast	WHC	1	1.09	0.141	9.32 ⋅ 10^−2^	0.986	25
		Slow	WHC	1	2.63	1.63	1.63	5.87	24
		Slow	WHC (*κ*_0_ = 10)	1	2.17	1.17	1.17	5.54	25
*T*_50_	Time at which within-host population size is half its final value	Prior	WHC	−4	−4.25	0.27	−0.254	7.05	25
		Fast	WHC	−4	−3.45	0.698	0.552	3.27	24
		Slow	WHC	−4	−1.42	2.58	2.58	3.86	16
		Slow	WHC (*κ*_0_ = 10)	−4	−1.71	2.29	2.29	4.68	23
*S*	Ratio of final within-host population size to size at infection	Prior	WHC	55.6	25.1	0.547	−0.549	1.53	25
		Fast	WHC	55.6	41.5	0.3	−0.253	1.08	23
		Slow	WHC	55.6	41.4	0.255	−0.255	1.33	23
		Slow	WHC (*κ*_0_ = 10)	55.6	41.5	0.253	−0.253	1.09	25

^1^ Molecular clock rates were not estimated for runs on the slow clock dataset

^2^ Infectious periods were drawn from a normal distribution with the “actual values” given here as mean and standard deviation. Error and bias were, however, calculated using the mean and standard deviation of the actual set of estimated periods from each simulated epidemic.

### Analysis of sequences from the 2003 H7N7 avian influenza outbreak in the Netherlands

**Fig 5:**
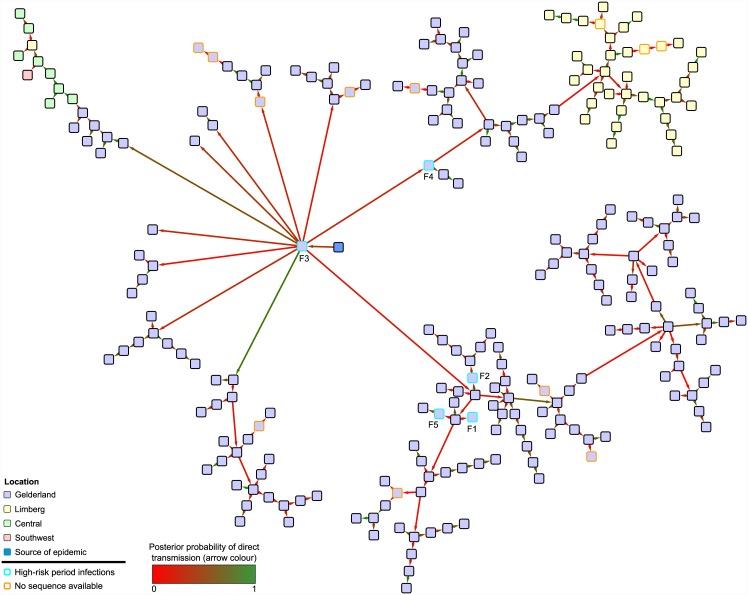
Maximum parent credibility transmission tree for the H7N7 outbreak. Nodes represent farms and are coloured by geographical region. Arrows represent direct transmissions and are coloured by the posterior probability of this particular direct infection. The cyan-bordered nodes, which are also labelled with farm ID numbers from previous literature [18], are were detected during the “high-risk” period before the implementation of control measures. Orange-bordered nodes are farms for which no sequence was available.

The MPC transmission tree can be seen in Fig 5. It can be seen that most of the inferred transmissions have a quite low posterior probability. In fact, if we were to use a posterior probability threshold of 0.5 to infer transmissions, we would draw conclusions about only 90 farms (37.3%), with this dropping to 24 (10%) for a threshold of 0.8, and 9 (3.7%) for a threshold of 0.9. None of the five “high-risk” farms met the 0.5 threshold, although the posterior probability that the index case was among these five was 0.62 compared to the prior probability of 0.17. This lack of resolution is the reason why in the MPC tree the presumed index farm F1 is not correctly identified, and why, while it and the other five high-risk period farms are close together at the start in the transmission chain, they are intermingled with other infected farms identified early in the epidemic. The posterior median date of the first infection was the 19th February, 2003, nine days prior to detection, with the 95% HPD ranging from the 16th until the 21st. This is somewhat later than previous estimates [36, 46]. The orange-bordered nodes in the tree are the twelve farms for which no sequence is available. Notably, the procedure placed them amongst their geographical neighbours. S2 Fig is the MCC phylogeny, with branches coloured by individual farm. It should be noted that the branch colourings in this figure do not reflect a history of the epidemic that is particularly representative of the posterior sample of transmission trees; they are simply the colourings of the phylogeny from the posterior with the highest clade credibility.

Table 6 summarises the parameter estimates. Of note, while we used an extremely informative prior distribution with a mean of two days on the length of the latent period, the estimate was still considerably shorter (posterior median 1.47 days, 95% HPD 1.26–1.69). The estimate of the mean infectious period in the low-risk period did not deviate greatly from the prior expected value of 7.3 (posterior median 7.52 days, 95% HPD 7.02–8.05). The posterior distribution for the mean during for the high-risk period, however, actually had a smaller median at 6.19 days (95% HPD 4.57–7.65), considerably shorter than the prior expected value of 13.8. On the other hand, the median infectious period of the index case (whichever it was in each MCMC state) was 12.7 days (9.59–16.7). Compared to the prior expected value of the precision of the distribution in both high-risk and low-risk periods of 0.263 days^−2^, the estimated precisions were lower, with posterior medians of 0.167 (6.1 ⋅ 10^−2^-5.41)days^−2^ for the high-risk period and 8.03 ⋅ 10^−2^ (6.28 ⋅ 10^−2^-0.105)days^−2^ for the low-risk period. The parameters of the within host population function suggested that the effective size of the infected population rose very quickly towards its asymptotic value, achieving values extremely close to it within a day or so. If the median estimates were used, this asymptotic population size was 10.6 times the value at the point of infection. While this behaviour would not seem to reflect the likely course of an epidemic within a flock, it should be remembered that the within-farm model was extremely simplistic for this example. As the analysis in here lacks data on uninfected susceptibles, the estimated parameters of the transmission kernel here differ considerably to the maximum likelihood figures from Boender et al (which were, in our notation, *b* = 2 ⋅ 10^−3^ days^−1^, *α*_1_ = 2.1 and *α*_2_ = 1.9) since the authors of that paper had access to data about every farm in the Netherlands.

**Table 6:** Estimates of parameters from the H7N7 outbreak, posterior median and 95% HPD interval.

Parameter	Symbol	Median value (95% HPD)
Transmission kernel parameters	*α*_1_	1.72 (1.53, 1.95)
	*α*_2_	0.652 (0.341, 1.03)
Unmodified transmission rate	*b*′	0.124 /day (5.39 ⋅ 10^−2^, 0.218)
Within-farm population growth rate	*r*	6.99 /day (4.61, 9.88)
Time before infection time at which *N*_*e*_ achieves half its final asymptotic value	*T*_50_	-0.321 days (-0.441, -0.22)
Latent period	*P* ^lat^	1.56 days (1.34, 1.8)
Mean infectious period (high-risk period)		6.19 days (4.57, 7.65)
Standard deviation of infectious periods (high-risk period)		2.45 days (0.43, 4.05)
Mean infectious period (low-risk period)		7.52 days (7.02, 8.05)
Standard deviation of infectious periods (low-risk period)		3.53 days (3.09, 3.99)
Mean molecular clock rate		2.78 ⋅ 10^−5^ subs/site/day (2.34 ⋅ 10^−5^, 3.25 ⋅ 10^−5^)
Standard deviation of molecular clock rates		1.34 ⋅ 10^−5^ subs/site/day (2.24 ⋅ 10^−6^, 2.40 ⋅ 10^−5^)
Transition/transversion ratio, positions 1+2		7.22 (4.78, 9.99)
Transition/transversion ratio, position 3		9.03 (5.56, 13.5)
Gamma shape parameter for between-site rate variation, positions 1+2		4.27 ⋅ 10^−2^ (1.02 ⋅ 10^−3^, 9.32 ⋅ 10^−2^)
Gamma shape parameter for between-site rate variation, position 3		0.231 (1.08 ⋅ 10^−3^, 0.677)
Nucleotide frequency, adenine (A)		0.333 (0.321, 0.346)
Nucleotide frequency, cytosine (C)		0.188 (0.178, 0.198)
Nucleotide frequency, guanine (G)		0.249 (0.238, 0.26)
Nucleotide frequency, uracil (U)		0.230 (0.219, 0.24)
Relative clock rate parameter, positions 1+2		0.853 (0.763, 0.939)
Relative clock rate parameter, position 3		1.29 (1.12, 1.47)

While properties of epidemics such as reproduction numbers are not readily derived from the parameters of a model of this type, they can be estimated post-hoc. For example, the posterior median number of farms infected by the index farm in this epidemic, which is analogous to the basic reproduction number (for farms) *R*_0_, was 7 (3–11). We also calculated, for each day in the epidemic, the mean number of farms subsequently infected by a farm infected on that day, which is equivalent to the effective case reproduction number *R*; the posterior distribution of this is summarised in S3 Fig. It should be noted the high values of *R* towards the start of the timeline come only from MCMC states for which the date of the index case was earlier than the median date of the 19th February; for many states no epidemic was present at that time and hence there were no infections to contribute to the calculation.

## Discussion

We provide here a novel method for simultaneous reconstruction of both phylogenies and transmission trees, fully incorporated into the existing BEAST package. Being part of an established package has the advantage that users of our method have access to existing models and methods for, for example, relaxed molecular clocks, ancestral sequence reconstruction, coalescent population models, and marginal likelihood estimation, without the need for extra programming work. The prior probability decomposition outlined above is also very flexible, allowing for many different distributions of infectious periods and models of spread between hosts.

The framework here builds primarily on the previous work of Didelot et al. [10] and Ypma et al. [8] combining the co-estimation of both trees from the latter with the internal node annotation of the former. The “colours” of Didelot et al correspond to our partition elements. (The earlier work of Cottam et al. [5] took a similar approach but insisted that each node was in the same partition element as one of its children, which makes the implicit assumption that lineages split at transmission as it is then impossible for any lineage to exist in a host if it is not the ancestor of a tip taken from that host.) We have extended this annotation to allow more than one tip to come from the same host, under the assumption that the host was infected only once. (If this may not be true, it might be more appropriate to treat the two introductions as separate “hosts”, particularly in an agricultural scenario.) This would be of use in, for example, the study of HIV, where multiple samples are often taken from the same patient over the course of treatment [47]. Our infection branch move serves the same purpose as the single move described by Didelot et al. but takes a rather different approach. The main difference is that it is a change to the tree partition, which may indirectly change an infection date, rather than to the infection dates themselves. Direct changes to infection dates in our framework are constrained to be those that cannot change the transmission tree, as they modify just the *q*_*i*_s. Other differences are that our version makes only moves that respect the partition rules (and hence the proposal never violates them) and makes no assumption that hosts cease to be infectious at any point (which is left as a job for likelihood calculations).

The work of Ypma et al. [8] has now been placed fully in the framework of modern phylogenetic inference. That paper treated every within-host phylogeny as a separate entity to be modified individually. For compatibility with existing packages such as BEAST, which estimate a single tree, it is more convenient to partition that tree instead. We also provide a more formal mathematical exploration of the properties of the joint space (see S1 Text), demonstrating that it is impossible for an MCMC procedure to fully explore the space of transmission trees without letting the phylogeny vary if the latter has more than two tips, and also that varying the phylogeny does allow the algorithm complete access. Finally, we show that this is indeed true in our case, by demonstrating irreducibility of our Markov chain. The move modifying the transmission tree proposed by Ypma et al. is most similar (although not identical) to our type B Wilson-Balding proposal, but irreducibility is not proven in the paper; the partition structure that we propose makes this much easier to show.

Exploration of the space of both trees is important, as the short timespan of phylogenies from epidemics and outbreaks often results in phylogenies that are not particularly well resolved, and as a result, a two-step procedure such as that of Didelot et al. or Cottam et al. may make it impossible to infer many plausible transmission histories. While in some circumstances access to the full space of transmission trees may not be necessary because some are very implausible (it is unlikely, for example, that the last host in an outbreak lasting months was infected by the first), which trees are implausible will vary greatly depending on the nature of the pathogen. It would be reasonable to rule out direct transmission between two individuals if their infection dates were separated by years if the pathogen was influenza, but not if it was HIV. Therefore, we considered it important, in designing a method intended to be general and flexible, to allow access to every single transmission tree. A simultaneous procedure such as this is to be preferred to the option of running a separate fixed-tree analysis on each of a set of trees from a Bayesian posterior sample for reasons of computational time, and because the fixed trees may have been estimated using an inappropriate model (see below).

The extent to which a fixed phylogeny constrains the space of transmission trees, or indeed vice versa, is a mathematical problem which is worthy of investigation. For a fixed phylogeny with three tips, 5 out of 9 transmission tree are possible (a proportion of 0.56); for four tips, it is either 12 or 13 out of 64 (0.19 or 0.2) (see Figure S1.1 in S1 Text); the total number of transmission trees increases faster than the total number of partitions of the phylogeny. As the partition count varies with the phylogenetic topology, there is no simple expression for the former based simply on the tip count of the latter, and analytical work to formally describe the relationship would be instructive. The opposite question, by how much the set of potential phylogenetic topologies is constrained if the transmission tree is known, also arises. While every transmission tree is possible in the joint framework presented here, the imposition of a transmission model certainly has a constraining effect on the credible set of phylogenies, as we saw when we compared the number of clades in the credible sets between the WHC and skyride slow clock results. The lack of difference between the two for the fast clock results is due to the fact that the genetic data alone is enough to largely resolve the phylogeny in that case and the reconstruction needs no additional help from the population model.

We found here that the WHC method was superior in estimating both the topological structure of the phylogeny and its node heights than an analysis using the GMRF Skyride tree prior; the latter, which assumes all lineages belong to a single, freely-mixing population, is amongst the most frequently employed current methods for the reconstruction of time-resolved phylogenies. This adds weight to the concerns we expressed in the Introduction about two-step methods that use a phylogeny or set of phylogenies estimated by another method as input for epidemiological reconstruction; the assumptions under which those trees were made may violate the population model that the epidemiological inference is using, and as a result they may not be accurate. As we have here developed a more accurate tree prior for an epidemic situation, we would recommend that the WHC be used for reconstruction of the phylogeny of suitable datasets even if the transmission tree is not of interest.

A frequent concern surrounding analyses of this sort has been the question of unsampled hosts or clinical cases. Some progress has been made in dealing with this issue recently in the non-phylogenetic methods [4, 11], and Numminen et al. [48] outlined a novel two-step, importance sampling method for the investigation of transmission trees using potentially sparsely sampled data based on a fixed, maximum-parsimony phylogeny. We go some way to addressing this problem in a one-step process because, as demonstrated, our method can include epidemiological information for known clinical cases for which no sequence is available. Scenarios of this sort, indeed, provide another reason to prefer a one-step approach; as a standard phylogenetic analysis is unaware of any epidemiological information other than dates of sampling, it has no information to use in placing a noninformative sequence in the tree. The position of a corresponding tip in a fixed phylogeny used as input for a two-step method will be effectively random. Our method can, instead, use epidemiological data such as the location of the case, as well as a prior or hyperprior on the time from infection to noninfectiousness, to place these with more certainty. It can be seen from the reconstructed transmission tree (Fig 5) from the H7N7 epidemic that the farms for which no sequence was available are placed amongst their geographical neighbours, which would be expected unless there was a particular reason to believe otherwise.

This is obviously not a complete solution to the problem; more challenging is the issue of the identification of unknown unsampled hosts in the transmission chain, and the quantification of the number of them. This is the principal limitation of this method, and further work is needed to address it. Two approaches have been suggested previously [10, 11], both of which could be accommodated as a modification to the WHC. The first is to create a pool of unsampled hosts, of variable size, and use reversible-jump MCMC [49] to add and subtract from it. Internal nodes in the phylogeny can then be assigned to elements of this set, obeying the rules about connectedness but disregarding that about each partition element containing a tip. The second is to allow hosts to “indirectly” infect others even after they ceased to be infectious. The assignment of a node to a particular partition element would no longer indicate that the lineage represented by that node was actually present in the host represented by the tip in the same element, but just that it was infected by that host before it entered any other sampled host. We suggest a third option, which is to allow the assignment of internal nodes to no host at all. The mathematical framework would require modification; for example, an expression would be needed for the probability of the infection of a host from an unknown source.

The assumption that transmission is a complete bottleneck is hard to relax, as one of the fundamental principles of the correspondence between transmission trees and partitions, that the nodes in a partition element form a connected subtree of the whole phylogeny, must then be discarded as the common ancestral node of two nodes in the same host may be outside that host. The realism of this assumption is often unclear and will vary from pathogen to pathogen; while the bottleneck has been found to be quite loose for individual-to-individual transmission of influenza [50], this may be less true when, as in our example, transmission is between farms [18]. For other organisms, such as HIV [51] and hepatitis C virus [52], it has been found that the number of transmitted variants is usually very small between individuals.

The assumption that the parameters of the three models are independent of each other is a simplification which could be relaxed in subsequent work. Potentially, all three could interact: within-host dynamics are likely to affect both the infectiousness of a particular host and the duration of its infection, and particular mutations may modify the behaviour of the infection both within and between hosts. The assumption that mutation is a neutral process is quite standard in phylogenetics, but there is considerable scope for work which instead accounts for selection.

Treating the infection status of a host upon examination as part of the data, rather than as background information, simplifies the mathematics surrounding infectious periods while allowing multiple examinations of the same host at different times. It has other consequences. On the positive side, it opens up the possibility of including the results of genuinely negative examinations as data, in a way that does not involve adjusting individual prior probabilities for infection times. Such a negative examination must be as near as possible to conclusive, however; the absence of clinical symptoms is certainly not sufficient. On the negative side, it means that an algorithm to sample from the *prior* probability distribution must be able to vary the number of tips in the tree, a very non-standard procedure which is not implemented currently in BEAST or any other commonly-used package. In addition, the assumption that examination does not disturb the infection will be in most cases a simplification; for many pathogens, positive examinations will result in clinical intervention which will shorten the time to noninfectiousness. The alternative described here whereby hosts become noninfectious upon examination avoids both these issues. If this too is regarded as unacceptable, the framework here could be adjusted to model the time from infection to first examination, rather than from infection to noninfectiousness.

As outlined in Methods, the parameters *b* and *ψ* determining between-host transmission cannot be estimated unless the set of uninfected susceptibles can be described. This may not be possible in many cases; in our H7N7 analysis we did not have such data and hence can only estimate *b*′ and *ψ*′, the values that they would take if no susceptibles remained at the end of the epidemic. One would expect transmission rates to be overestimated if there is no contribution from hosts that were never infected. If it is not feasible to acquire this information and these parameter are still of particular interest, then a method to estimate the contribution of the set of uninfecteds is needed.

The WHC method was very successful in recapturing the epidemiological parameters of the simulations where sequences were generated by a fast clock, in which case the level of genetic diversity was such that there was little uncertainty in the phylogeny. Moving to a more realistic level of diversity decreased the accuracy of estimates considerably, and this illustrates the importance of using existing information, be it genetic or epidemiological, when configuring an analysis of this type. In particular, the results of the simulation analysis showed a clear bias towards underestimating the infectious periods of hosts. The reason for this is that the kind of within-host phylogenies that maximise the probability expressions Eqs (1) and (2) with an increasing within-host population are those that have only short periods in which the tree has more than one lineage, and where those short periods are close to the time of infection. The probability each such phylogeny is therefore increased, all else being equal, by moving the infection time towards the tips. This phenomenon seems similar to that which arises from the specification of “calibration densities” [53] for the root height in a standard time-tree analysis where that prior distribution conflicts with the root height that would be expected from the priors on the coalescent parameters, although as the WHC considers multiple coalescent trees with variable tip dates, the situation is considerably more complicated. The interaction between within- and between-host models is clearly complex and could motivate analytical work to find variants in which the effective priors do not interfere with each other in this way.

In any case, the bias in estimation of infectious periods is overwhelmed by sufficient genetic data (as in the fast clock dataset) and can be largely mitigated by placing a suitably informative prior on the length of infectious periods. A clear implication of this is that genetic data should not be relied upon to estimate infectious periods on their own if other information is available to inform such a prior. (We note that this bias does not affect estimation of who infected who, as the accuracy of transmission tree reconstruction did not significantly change when we changed the prior on *μ*_inf_.) In a similar vein, the lack of genetic diversity in the slow clock dataset meant that in some cases the molecular clock rate itself could not be reliably estimated and had to be fixed to its known value. In an actual epidemic situation it would seem perfectly reasonable to do this, using a rate derived from older data, unless it was clear that the pathogen in question was novel.

The concerns about the use of uninformative priors on infectious periods led us to use informative distributions taken from previous literature in the H7N7 analysis, and we continued the practice from the simulations of using highly informative priors on latent periods as preliminary work had shown that these tended not to be well estimated using uninformative priors. Some analysis results nevertheless deviated from what would be expected under the prior distributions; in particular the estimated mean infectious period during the high-risk period, and the estimated latent period, were both considerably smaller than their prior expected values. While it is possible that these underestimates are at least partly the result of the bias that was noted in the simulations, the difference is considerably more extreme than anything observed there. While our analysis agrees that the infectious period in the index case may have been around two weeks, the genetic data seems to suggest, contrary to previous work [36, 37], this was not the case for the remaining high-risk farms and that they may have been infectious for a shorter time than the low-risk period farms. This suggests that the infection was present within the index farm for a considerable time before transmission began to occur. The estimated shorter periods in the other high-risk farms may be due to increased surveillance in nearby facilities once the index infection was discovered. In the case of the latent period, the MCMC never actually sampled a value of two days or more at all, which previous work had assumed *a priori* as its length. While it is true that the assumption of a single latent period for all farms is a considerable simplification, this still suggests that the phylogeny is simply unable to accommodate a situation where all latent periods are of two days or greater. This analysis also suggested much greater variation in the lengths of infectious periods than had been previously estimated, in the low-risk period at least. These three observations suggest possible insights that genetic data can provide have not been apparent in traditional analyses.

The other model parameters that are not well recovered in the slow clock simulation dataset are those of the within-host coalescent process. This paper has concentrated on the between-host model, and if the WHC method it is to be used to investigate within-host dynamics in detail then further work is needed. It may be that the situation is improved if multiple sequences are taken from the same host. The estimated parameters of the logistic growth function for H7N7 should certainly not be overinterpreted, for several reasons. First, it is a gross oversimplification to assume that the infected population of each farm grew according to the same function, especially when the farms infected in this epidemic ranged from hobby farms to large agricultural facilities. Secondly, the “effective number of infections” will differ from the true size of the infected population due to the violation of assumptions made in the coalescent process. While the WHC is designed to deal with violations of the assumption of homogeneous mixing of lineages that in fact infect separate hosts in an epidemic, lineages would not be expected to mix freely within farms (or indeed host organisms) either. It has also been shown that if the population in a coalescent model is treated as being made up of infected individuals, the relationship between the effective population size and prevalence is not as straightforward as might be assumed [17, 54]. Lastly, logistic growth may be too simplistic a model of the infected population. It was picked here because it is clearly a better fit to growth within a farm than a constant population size or exponential growth, but true dynamics are no doubt more complicated still.

Even the “slow clock” simulation dataset was intended to represent the full genome of influenza A, one of the most fast-evolving pathogens that is likely to cause an outbreak of this type. The resolution in the reconstruction of the transmission tree for the H7N7 outbreak could be increased if sequences for the remaining segments of the genome were available; consistently higher posterior probabilities for infectors were observed in the simulation analyses. As the short timescale of an epidemic already places a limit on the amount of information that can be gleaned from genetic data, we would suggest that resources be expended to sequence as much of the pathogen genome as possible in a situation of this sort.

In conclusion, what we have demonstrated here is both a new phylogenetic method for the analysis of genetic data taken from outbreaks and epidemics, and a new transmission tree reconstruction method. For phylogeny reconstruction we have developed a population model that is more realistic than the assumptions of freely-mixing lineages that are made in the most widely-used current methods. For transmission tree reconstruction, we have advanced the development of models that accommodate within-host diversity with a procedure that maintains the previously-noted correspondence between transmission trees and the annotation of internal nodes in a phylogeny while exploring the full space of phylogenies, which is required to allow access to the full space of transmission trees. As part of BEAST, it is publicly available (as of version 1.8.2), and compatible with any other model of interest that is implemented in that package. We hope that it will prove useful in future for researchers working on genetic analysis of outbreaks.

## Supporting Information

S1 Fig The effects of fixing the within-host model parameters on the reconstruction of the transmission tree. Each violin plot represents the density of a statistic over the 25 simulations; results come from analyses where the within-host model parameters were estimated and fixed for the fast and slow clock datasets. (A) posterior median of mean bias in estimation of infection dates. (B) posterior median of mean error in estimation of infection dates. (C) Posterior median proportion of hosts whose infector is correctly identified. (D) Proportion of hosts whose infector is correctly identified in the maximum parent credibility (MPC) transmission tree.
(EPS)

S2 Fig Maximum clade credibility phylogentic tree for the H7N7 outbreak. This is the actual sampled tree with highest clade credibility from the posterior set; branch lengths have not been adjusted. Branches are coloured by farm; colour changes along branches reflect infection events.
(EPS)

S3 Fig Reconstruction of the change in effective case reproduction number over the H7N7 epidemic. The black line represents the posterior median value of the mean number of secondary cases caused by a case infected during each day, the blue lines the upper and lower bounds of the 95% highest posterior density interval.
(EPS)

S1 Text Supplementary methods. Full details of the correspondence between transmission trees and partitions of the nodes of a phylogeny, and of the MCMC proposals.
(PDF)

S2 TextSupplementary methods 2.Derivation of the probability $p(N,T^{\text{inf}},T^{\text{trans}},T^{\text{end}}|b,\phi ,\rho ,\lambda ,L)$.(PDF)

S1 Data BEAST XML files for simulated data. BEAST input file for all WHC analyses of all simulated datasets.
(ZIP)
