# Supplementary material for: Epidemic Reconstruction in a Phylogenetics Framework: Transmission Trees as Partitions of the Node Set
Source: PLoS Comput Biol. 2015 Dec 30;11(12):e1004613. doi: 10.1371/journal.pcbi.1004613 (PMC4701012; doi:10.1371/journal.pcbi.1004613)
Supplement: S1 Text — Full details of the correspondence between transmission trees and partitions of the nodes of a phylogeny, and of the MCMC proposals. (PDF) [file pcbi.1004613.s004.pdf]

Epidemic Reconstruction in a Phylogenetics Framework: Transmission Trees as Partitions of the Node Set

Supplementary Methods

Matthew Hall   Mark Woolhouse   Andrew Rambaut

S1.1 Introduction

The purpose of this supplemental document is to outline the mathematical basis for the observation that a MCMC procedure that samples from the space of partitioned phylogenies is in fact sampling simultaneously from the space of phylogenies and transmission trees, in more detail than was possible in the main paper. We begin with some fairly straightforward mathematics to formalise the correspondence between partitions and transmission trees, and then describe all the MCMC moves in full detail. We also prove that an MCMC chain using these moves to sample from the space of partitioned phylogenies is irreducible.

S1.2 Transmission trees as partitions of the node sets of phylogenies

Suppose the set of set of hosts infected in the epidemic is $\mathbf{A} = \{a_1, \ldots, a_N\}$ and the set of isolates is $\mathbf{B} = \{B_1, \ldots, B_M\}$. Let $f : \mathbf{B} \to \mathbf{A}$ be a map taking an isolate to the host it was sampled from

and assume f is surjective (which implies $M \geq N$). Suppose also that there was no reinfection or superinfection, and that transmission is a complete bottleneck: only a single genetic variant enters the newly infected host upon transmission. Let $\mathcal{G}$ be a phylogeny describing the ancestral relationship of the members of $\mathbf{B}$, with branch lengths in units of time. It consists of two components:

- A rooted, binary tree G with a set $\mathbf{E}_G$ of M tips labelled, via a function g, with the elements of $\mathbf{B}$ and a set $\mathbf{I}_G$ of $M-1$ internal nodes. Let $\mathbf{N}_G = \mathbf{E}_G \cup \mathbf{I}_G$ be the complete set of nodes. Let $\mathbf{G}_\mathbf{B}$ be the set of all such trees with tips allocated to isolates via g and isolates allocated to hosts via f. The map $d = f \circ g : \mathbf{E}_G \rightarrow \mathbf{A}$ labels each tip with a host. Call G the *topology* of the phylogeny.
- A length function $l : \mathbf{N}_G \rightarrow (0, \infty)$ that takes each non-root node of G to the difference in calendar time (in arbitrary units on a forwards timescale) between the time of the event represented by that node and the time of the event represented by its parent. The event represented by an element u of $\mathbf{E}_G$ (a tip) is the sampling of the isolate from the host corresponding to u's label; the event represented by an element v of $\mathbf{I}_G$ (an internal node) is the coalescence of the two lineages represented by v's two child branches; this occurs at the TMRCA of those lineages. In contrast to the convention in most phylogenetic methods, we do indeed define a nonzero $l(r)$ for the root node r of G. Its value is largely arbitrary, but it must be greater than any plausible value for the time between the event (generally a coalescence) represented by r and the infection event that seeded the entire outbreak.

The length function l allows us to also define a height function $h : \mathbf{N}_G \rightarrow [0, \infty)$ that takes each node to the difference in time between the event represented by that node and the time at which the last isolate was sampled. This map defines a backwards timeline for events on the whole tree whose zero point is the latter time.

A transmission tree on $\mathbf{A}$ is a rooted, directed tree with M nodes labelled with the elements of $\mathbf{A}$. If $\mathcal{N}$ is such a tree, it can be thought of as a map $\mathcal{N} : \mathbf{A} \rightarrow \mathbf{A} \cup \emptyset$ taking each host a_i to its infector or to

$\emptyset$ if a_i is the first host, and we will use this notation henceforth. Let $\mathbf{\Pi_A}$ be the set of all transmission trees on $\mathbf{A}$. ($\mathbf{\Pi_A}$ has cardinality N^{N-1} by Cayley's formula, as there are N^{N-2} such trees and N choices of root for each.) Take G to be a topology as above, describing the ancestry of $\mathbf{B}$ without meaningful branch lengths. We are interested in the set of transmission trees in $\mathbf{\Pi_A}$ that are consistent with the ancestry represented by G.

It is quite obvious (see figure 1 of the main text) that if each node in G is mapped to the host in which the corresponding pathogen lineage was present, then the transmission tree is known. We now wish to formally establish this link. This will allow us to stop dealing with the transmission tree as a separate entity, and instead treat it as a function applied to the internal nodes of G.

Definition S1.2.1. Let $\mathbf{\Omega}^{G,d}$ be the set of partitions of $\mathbf{N}_G$ such that:

- If $\mathcal{P} \in \mathbf{\Omega}^{G,d}$ and $p \in \mathcal{P}$ (such a p being a subset of $\mathbf{N}_G$), then the subgraph of G induced by p (the subgraph having node set p and retaining all edges adjacent to two elements of p) is connected.
- If $\mathcal{P} \in \mathbf{\Omega}^{G,d}$ and $p \in \mathcal{P}$, then $|\mathbf{E}_G \cap\ p| \geq 1$ and $|\{d(u) : u \in \mathbf{E}_G \cap\ p\}| = 1$. In other words, each p contains tips which correspond to isolates taken from one, and only one, host.

Definition S1.2.2. $\mathbf{\Omega}^{G,d}$ may be empty if d is such that no partitions of this type exist. If it is not, say G is *compatible* with d.

For a fixed G, define a map $c : \mathbf{A} \to \mathbf{N}_G$ taking $a_i \in \mathbf{A}$ to the most recent common ancestor of all tips $u \in \mathbf{E}_G$ with $d(u) = a_i$. If $|d^{-1}(a_i)| = 1$, i.e. only one isolate was taken from a_i, $c(a_i)$ is a tip.

Proposition S1.2.3. *G is not compatible with d if and only if there exist hosts $a_i, a_j \in \mathbf{A}$ such that $|d^{-1}(a_i)| \geq 2$ and $|d^{-1}(a_j)| \geq 2$ and either $c(a_i)$ is an ancestor of $c(a_j)$ but there exists $v \in d^{-1}(a_i)$ ($d^{-1}(a_i)$ being the set of tips corresponding to isolates taken from a_i) such that v is a descendant of $c(a_j)$, or $c(a_j)$ is an ancestor of $c(a_i)$ but there exists $v \in d^{-1}(a_j)$ such that v is a descendant of $c(a_i)$.*

Proof. For "if", if $c(a_i)$ is an ancestor of $c(a_j)$ and there exists such a v, then if $\mathcal{P}$ is a partition such that $p \in \mathcal{P}$ contains the whole of $d^{-1}(a_j)$ and the subgraph induced by p is connected, then it must

contain $c(a_j)$, and thus the subgraph induced by a partition element containing $c(a_i)$ and v cannot be connected as a path from one to the other must intercept $c(a_j)$. Likewise if there is a $p \in \mathcal{P}$ containing $d^{-1}(a_i)$ such that the subgraph induced by p is connected, then it must contain $c(a_j)$ and hence any partition element containing $d^{-1}(a_j)$ cannot.

For "only if", if no such hosts exist, put a partial order on the $c(a_i)$ for all i such that $c(a_i) \preceq c(a_j)$ if $c(a_i)$ is a descendant of $c(a_j)$. Permute the $c(a_i)$ into a sequence $U = \{c(a_{o(1)}), \ldots, c(a_{o(N)})\}$ such that $o(i) \leq o(j)$ if $c(a_{o(i)}) \preceq c(a_{o(j)})$. Build a partition $\mathcal{P}$ by moving through U, assigning each $c(a_{o(i)})$ and each descendant of $c(a_{o(i)})$ that is not $c(a_{o(j)})$ or a descendant of $c(a_{o(j)})$ for $j < i$ to a new partition element. At the end of the process, perform a post-order traversal of the tree, assigning any remaining unassigned nodes encountered to a partition containing one of their children. It is clear that at the end, $\mathcal{P}$ has the required N elements. By construction, all nodes assigned to the same partition element form a connected subgraph of G, so it remains only to check the second half of definition S1.2.1. Suppose there exists an a_i such that there is a tip $v_i \in d^{-1}(a_i)$ which was not assigned to the same partition element as $c(a_i)$. This implies that there exists an a_j such that $c(a_j)$ is a descendant of $c(a_i)$ and v_i is a descendant of $c(a_j)$, which is the only way v_i would not have been assigned to $c(a_i)$'s element. As neither $c(a_i)$ nor $c(a_j)$ can be a tip, $|d^{-1}(a_i)| \geq 2$ and $|d^{-1}(a_j)| \geq 2$, and a_i and a_j are the kind of hosts we assumed did not exist. So all tips in each $d^{-1}(a_i)$ are assigned to the same element, the one containing $c(a_i)$, and this set of tips has size at least one since $c(a_i)$ is a common ancestor of at least one node. □

Corollary S1.2.4. *If $N = M$ or $N = M - 1$ then all phylogenetic trees G are compatible with d.*

Proof. In this case all, or all but one, of the $c(a_i)$ are tips and thus have $|d^{-1}(a_i)| = 1$ □

From now on, assume we are working with a G that is compatible with d. For $\mathcal{P} \in \mathbf{\Omega}^{G,d}$, extend d to a map $d_{\mathcal{P}} : \mathbf{N}_G \to \mathbf{A}$ that takes each node of G to the host of the tips that are in the same element of $\mathcal{P}$ as itself. For each $a_i \in \mathbf{A}$, let $H_{\mathcal{P},i}$ be the subtree of G induced by the set $d_{\mathcal{P}}^{-1}(a_i)$. Because $H_{\mathcal{P},i}$ is connected, it has a single root node. Define a second map $e_{\mathcal{P}} : \mathbf{A} \to \mathbf{N}_G$ taking each a_i to this root

node. For brevity write $s_i = e_{\mathcal{P}}(a_i)$. All s_i have a parent s_iP in G, except for the root r of G (which must be the root of one such subtree).

We interpret a partition $\mathcal{P}$ in $\mathbf{\Omega}^{G,d}$ such that the lineages represented by all nodes in $\mathcal{P}$ were present in the single host that all tips in $\mathcal{P}$ were sampled from. Then $\mathcal{P}$ can be taken to a transmission tree by using $d_{\mathcal{P}}$ to annotate each node u of G with that host. We then know who infected whom; infection events occur along branches of G whose start and end nodes are in different elements of $\mathcal{P}$. The preimage of $a_i \in \mathbf{A}$ under $d_{\mathcal{P}}$ is the set of nodes of $H_{\mathcal{P},i}$. The rules by which partitions are defined correspond to the assumptions about the epidemic. The connectedness requirement implies no reinfection or superinfection (if a host could experience multiple infections then the subtree induced by its partition element would not be connected) and also that transmission is a complete bottleneck (or else the two child lineages of an internal node could both be transmitted to the same host at the same time, and again the induced subtree would be disconnected). The requirement that all partition elements contain a tip is a result of the surjectivity of f (every host is sampled at least once). Proposition S1.2.3 shows that if G is not compatible with f, then the assumption of no reinfection or superinfection must be violated due to the placement of tips from the the same host in the phylogeny.

To formalise the correspondence, we construct a map $z : \mathbf{\Omega}^{G,d} \to \mathbf{\Pi}_{\mathbf{A}}$ such that if $\mathcal{P} \in \mathbf{\Omega}^{G,d}$ and $a_i \in \mathbf{A}$,

$$z(\mathcal{P})(a_i) = \begin{cases} d_{\mathcal{P}}(s_iP) & s_i \neq r \\ \emptyset & s_i = r. \end{cases}$$

In other words, $z(\mathcal{P})$ returns the infector of a_i if the partition is $\mathcal{P}$.

Proposition S1.2.5. *For $\mathcal{P} \in \mathbf{\Omega}^{G,d}$, the directed graph T given by drawing an edge from $z(\mathcal{P})(a_i)$ to a_i for all $a_i \in \mathbf{A}$ is a directed tree, and if r is the root of G, the directionality is consistent with $d_{\mathcal{P}}(r)$ being the root of T.*

Proof. For the first part, we must show that the underlying undirected graph T' of T is connected and has no simple cycles. Suppose that it has a simple cycle passing through $n > 1$ distinct nodes $a_1, \ldots, a_n$.

The construction of T will never give a node with indegree greater than 1 (as every host is infected once only), so this cycle must be directed in T; without loss of generality suppose the sequence $a_1, \ldots, a_n$ follows this directionality. Then $z(\mathcal{P})(a_k) = a_{k+1}$ for all $1 \leq k \leq n-1$ and $z(\mathcal{P})(a_n) = a_1$. If $i \geq 2$, $H_{\mathcal{P},i}$ is a subtree of G containing a root node s_i and the parent $s_{i-1}P$ of the root node of the subtree $H_{\mathcal{P},i-1}$; similarly $H_{\mathcal{P},1}$ contains s_nP. Since $H_{\mathcal{P},i}$ for each $i \geq 2$ contains a sequence of nodes, following the directedness of G induced by its root, running from s_i to $s_{i-1}P$, $H_{\mathcal{P},1}$ contains one running from s_1 to s_nP, and there is a directed link from each s_iP to s_i in G, the concatenation of all of these forms a simple cycle in G, contradicting the fact it is a tree.

For connectedness, again suppose $a_i \in \mathbf{A}$ and let $a_j = d_{\mathcal{P}}(r)$; r is the root of both $H_{\mathcal{P},j}$ and G. It may be that $a_i = a_j$. If not, the path in G from s_i to s_j intersects $n \geq 2$ elements of $\mathcal{P}$ whose members map under $d_{\mathcal{P}}$ to the hosts $a_{o(1)}, \ldots, a_{o(n)} \in \mathbf{A}$, where o is some permutation of $\{1, \ldots, N\}$ with $o(1) = i$ and $o(n) = j$. In particular it must pass through the root nodes of all these subtrees, $s_{o(1)}, \ldots, s_{o(n-1)}$, implying that $z(\mathcal{P})(a_{o(k)}) = a_{o(k+1)}$ for all $1 \leq k \leq n-1$. It follows that $(z(\mathcal{P}))^{n-1}(a_i) = a_j$; thus all elements of $\mathbf{A}$ are connected to a_j and furthermore to each other in T'. This also implies the existence of a directed path in T from a_j to any other a_i.

For the second part, $d_{\mathcal{P}}(r)$ has indegree 0 by construction, and we already have a directed path from $d_{\mathcal{P}}(r)$ to each $a \in \mathbf{A}$. As we have shown T is a tree, this is the only such path, hence the direction of all edges is away from $d_{\mathcal{P}}(r)$. □

Proposition S1.2.6. *z is injective.*

Proof. Suppose that there are two partitions $\mathcal{P}, \mathcal{P}'$ that have the same image under z, i.e. for all $a_i \in \mathbf{A}$, $z(\mathcal{P})(a_i) = z(\mathcal{P}')(a_i)$. If $\mathcal{P} \neq \mathcal{P}'$ then there exists some node u of G that has $a_i = d_{\mathcal{P}}(u) \neq a_j = d_{\mathcal{P}'}(u)$. It can be assumed that either u is the root of G or $d_{\mathcal{P}}(uP) = d_{\mathcal{P}'}(uP)$ for the parent uP of u (otherwise it is possible to move up G, towards the root, to find a new u for which this is true).

If u is the root of G, then it is the root of the subtrees $H_{\mathcal{P},i}$ and $H_{\mathcal{P}',j}$. This implies $z(\mathcal{P})(a_i) = \emptyset$ but $z(\mathcal{P}')(a_i) \neq \emptyset$ because $z(\mathcal{P}')(a_j) = \emptyset$ and only one element of $\mathbf{A}$ has image $\emptyset$ under $z(\mathcal{P}')$ since the

root of G is unique. So uP exists.

Let $a_k = d_{\mathcal{P}}(uP) = d_{\mathcal{P}'}(uP)$. First suppose $k \neq i$ and $k \neq j$. Then $z(\mathcal{P})(a_i) = a_k$. We show that $z(\mathcal{P}')(a_i) = a_k$ is not possible. Let v be any tip of G with $d(v) = a_i$. Now v is a descendant of u because u is the root node of the subtree $H_{\mathcal{P},i}$, and $H_{\mathcal{P},i}$ includes v. $\mathcal{P}'$ gives rise to another subtree of G, $H_{\mathcal{P}',i}$, all of whose nodes map to a_i under $d_{\mathcal{P}'}$. This $H_{\mathcal{P}',i}$ has a root node s'_i which is *not* u because $d_{\mathcal{P}'}(u) = a_j$. It must, in fact, also be a descendant of u; if it were not, $H_{\mathcal{P}',i}$ would not be connected as it would include a node v that was a descendant of u and nodes that were not. The parent s'_iP cannot have $d_{\mathcal{P}'}(s'_iP) = a_k$ because either a) $s'_iP = u$ and $d_{\mathcal{P}'}(u) = a_j$ by construction or b) $s'_iP \neq u$ and if $d_{\mathcal{P}'}(s'_iP) = a_k$ were true, the subtree induced by set of nodes that map to a_k under $d_{\mathcal{P}'}$ would not be connected because that set would include uP which is u's parent and s'_iP which is a descendant of u. Hence $z(\mathcal{P}')(a_i) \neq a_k$.

So without loss of generality suppose $k \neq i$ but $k = j$. Again $z(\mathcal{P})(a_i) = a_k$. Recall that $d^{-1}(a_k)$ is the set of tips of G that map to a_k under d and by extension $d_{\mathcal{P}}$ and $d_{\mathcal{P}'}$. No elements of $d^{-1}(a_k)$ are descendants of u. If any were, then $H_{\mathcal{P},k}$, the subgraph of G induced by the nodes mapped to a_k by $d_{\mathcal{P}}$, would be disconnected because u, which maps to a_i, would be absent. This implies that there is a node w of G, either a descendant of u or u itself, which maps to a_k under $d_{\mathcal{P}'}$ but neither of whose children wC_1 and wC_2 do. If $w = u$ then $d_{\mathcal{P}}(w) \neq a_k$ by construction. If $w \neq u$ and $d_{\mathcal{P}}(w) = a_k$, w would have an ancestor, u, which did not map to a_k under $d_{\mathcal{P}}$, and an earlier ancestor, uP, which did, breaking connectedness. This implies that $z(\mathcal{P}')(wC_1) = z(\mathcal{P}')(wC_2) = a_k$ but $z(\mathcal{P})(wC_1) = z(\mathcal{P})(wC_2) \neq a_k$.

□

For the next proposition, we need the following:

Lemma S1.2.7. *If $a_i, a_j \in \mathbf{A}$ and $\mathcal{N} \in \mathbf{\Pi_A}$ is a transmission tree in which a_i is an ancestor of a_j, then if $\mathcal{P} \in \mathbf{\Omega}^{G,d}$, $z(\mathcal{P}) = \mathcal{N}$, and u is a node of G with $d_{\mathcal{P}}(u) = a_j$, u has an ancestor v in G with $d_{\mathcal{P}}(v) = a_i$.*

Proof. Strong induction on the number n of intervening hosts between a_i and a_j in $\mathcal{N}$. If $n = 0$, this

is true by definition of u, as the node s_j is an ancestor of u and its parent maps to a_i. If the lemma is true for all $n \leq m$ and the set of intervening hosts has size $m+1$, let a_k be an arbitrary member of that set. The number of intervening hosts between a_k and a_j in $\mathcal{N}$ is less than $m+1$, so u has an ancestor v in G with $d_{\mathcal{P}}(v) = a_k$. The number of intervening hosts between a_i and a_k in $\mathcal{N}$ is also less than $m+1$, so v has an ancestor w in G with $d_{\mathcal{P}}(w) = a_i$. It follows that w is the ancestor of u that we need. □

Proposition S1.2.8. *z is not surjective for $N > 2$.*

Proof. If $N > 2$ then $M > 2$. Let $a_i, a_j, a_k \in \mathbf{A}$ be any three hosts. In G, let t_i, t_j, t_k be any three tips with $d(t_i) = a_i$, $d(t_j) = a_j$ and $d(t_k) = a_k$. These tips have a most recent common ancestral node u and two of them, without loss of generality t_j and t_k, have a most recent common ancestral node v which is a descendant of u. We show that there is no element of $\mathbf{\Omega}^{G,d}$ which will map to any member of $\mathbf{\Pi_A}$ in which any of the following are true:

- a_j is an ancestor of a_i, which is an ancestor of a_k.
- a_j is an ancestor of a_k, which is an ancestor of a_i.
- a_k is an ancestor of a_i, which is an ancestor of a_j.
- a_k is an ancestor of a_j, which is an ancestor of a_i.

Let $\mathcal{P}$ be a partition such that $z(\mathcal{P})$ is a transmission tree in which a_j is an ancestor of both a_i and a_k (if no such transmission tree exists, then surjectivity is instantly disproven). Now $d_{\mathcal{P}}(u) = a_j$. To see this, note that since u is an ancestor of t_j, if it does not map to a_j under $d_{\mathcal{P}}$ then neither do any of its ancestors, by connectedness. Nor do any descendants of the child of u which is not an ancestor of t_j and t_k, a set which includes t_i. All ancestors of t_i apart from u belong to one of those two categories. But this contradicts lemma S1.2.7 because t_i has no ancestor which maps to a_j under $d_{\mathcal{P}}$ despite the fact that a_j is an ancestor of a_i in $z(\mathcal{P})$.

Now t_i has no ancestor in G that maps to a_k under $d_{\mathcal{P}}$, because the node u breaks connectedness between t_k and any position that such a node could be. The contrapositive of lemma S1.2.7 then says that a_k is not an ancestor of a_i in $z(\mathcal{P})$. Similarly a_i is not an ancestor of a_k. An identical argument will show that if $z(\mathcal{P})$ is such that a_k is an ancestor of both a_i and a_k, a_i is not an ancestor of a_j nor vice versa.

□

Let the image of $\mathbf{\Omega}^{G,d}$ under z be $\mathbf{\Pi}_{\mathbf{A}}^{G,d} \subseteq \mathbf{\Pi}_{\mathbf{A}}$. The actual cardinality of $\mathbf{\Pi}_{\mathbf{A}}^{G,d}$ varies with the topology of G, which can be clearly seen in the case $M = 4$ and $N = 4$ (figure S1.1).

Proposition S1.2.6 states that no two partitions of the internal nodes of G correspond to the same transmission history; the set of partitions and the set of transmission trees that are actually possible if G is the correct ancestry are equivalent. Proposition S1.2.8 shows, however, that not every possible transmission tree on $\mathbf{A}$ actually corresponds to a partition of the nodes of a fixed G, except in the trivial case where there are only two hosts. If we are interested in exploring the complete space of transmission trees using this construction, the phylogenetic topology must be varied as well.

Let the set $\mathbf{\Omega} = \{\mathbf{\Omega}^{G,d} : G \in \mathbf{G_B}\}$ consist of all partitions of all phylogenies with tips labelled with $\mathbf{B}$ (via a map g) and $\mathbf{A}$ (via $d = g \circ f$). The map z can be extended to a map $Z : \mathbf{\Omega} \to \mathbf{\Pi}_{\mathbf{A}}$ in the obvious way.

Proposition S1.2.9. *Z is surjective. In other words, any transmission tree on $\mathbf{A}$ arises as a partition of some phylogenetic tree topology $G \in \mathbf{G_B}$.*

Proof. Let $\mathcal{N} \in \mathbf{\Pi}_{\mathbf{A}}$. Use the following procedure to construct an element of $\mathbf{\Omega}$. For all i, suppose m_i is the number of isolates taken from $a_i \in \mathbf{A}$ (in other words, $m_i = |d^{-1}(a_i)|$) and a_i has n_i children in $\mathcal{N}$. Consider the phylogeny of the lineages infecting the host a_i. This has $m_i + n_i$ tips (one for each sample taken and one for each lineage that was transmitted to another host) and hence $m_i + n_i - 1$ internal nodes. However, the n_i tips corresponding to onwards infections do not represent nodes in the

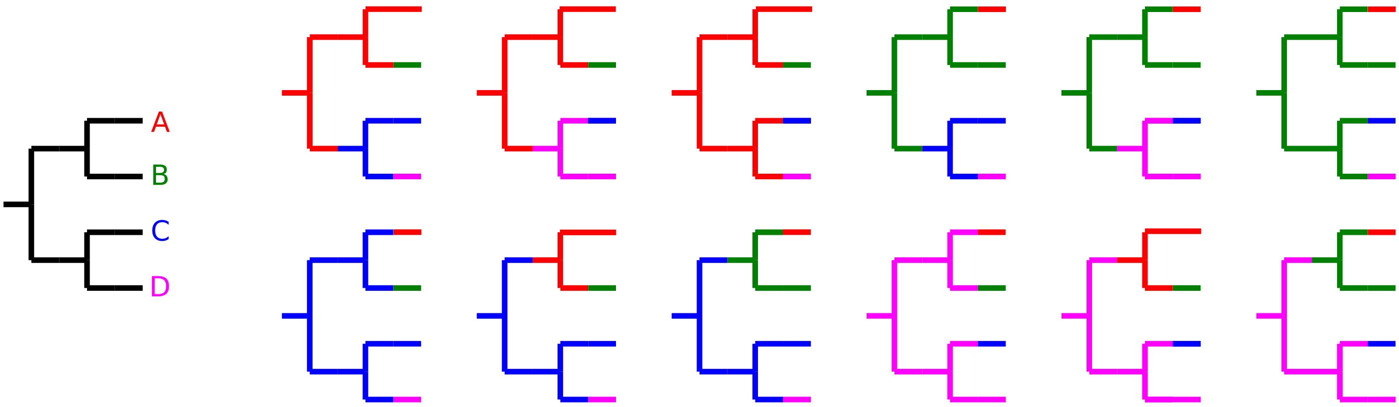

(a)

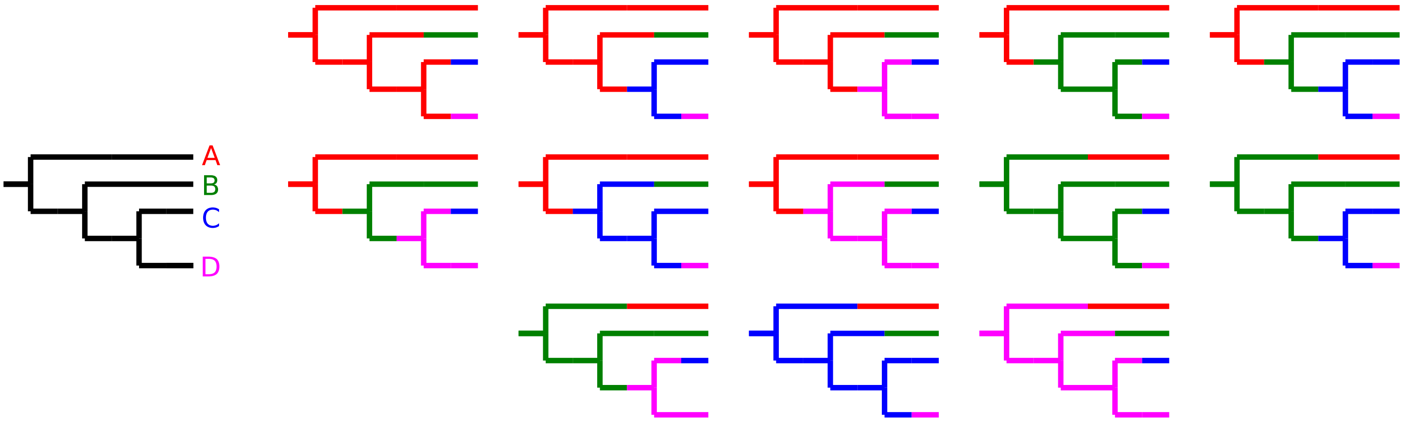

(b)

Figure S1.1: Illustration of the differing number of partitions of two phylogenies with the same tip count. a) the twelve valid partitions of the phylogeny ((A,B),(C,D)) for four hosts. b) the thirteen valid partitions of the phylogeny (A,(B,(C,D))) for four hosts

full phylogeny of the epidemic, so let $u^i_1, \ldots, u^i_{m_i}$ be nodes that are to represent sampling events (tips in the full phylogeny), and $v^i_1, \ldots, v^i_{m_i+n_i-1}$ be nodes to represent common ancestors.

Pick an arbitrary ordering of the children of each a_i in $\mathcal{N}$ and draw edges from each v^i_k to v^i_{k+1} and from v^i_k to v^j_1 where j and k are such that a_j is the kth child of a_i in the ordering. For each i, the nodes $v^i_1, \ldots, v^i_{n_i}$ now have two children each; $v^i_{n_i+1}, \ldots, v^i_{n_i+m_i-1}$ still have none. There are $m_i - 1$ of those, so they and $u^i_1, \ldots, u^i_{m_i}$ can be connected into an arbitrary binary tree with the former as internal nodes, the latter as tips, and $v^i_{n_i+1}$ (which is already connected to $v^i_{n_i}$ by an edge) as the root. When this has been performed for all i, call the full graph G. If $l \in \{1, \ldots, N\}$ is such that a_l is the root of $\mathcal{N}$, let the root of G be $v_{l,1}$.

It is clear that G is a rooted binary tree. Its tip set $\mathbf{E}_G$ consists of $u^i_1, \ldots, u^i_{m_i}$ for each i. Let g be any bijective map from $\mathbf{E}_G$ to $\mathbf{B}$ such that $f \circ g(u^i_j) = a_i$ for all i and j. For each i, the set of nodes $\{u^i_1, \ldots, u^i_{m_i}\} \cup \{v^i_1, \ldots, v^i_{m_i+n_i-1}\}$ forms, by construction, a connected subtree of G and contains a nonempty set of tips whose image under $d = f \circ g$ is of size one; hence this partition of the nodes of G is an element $\mathcal{P}$ of $\mathbf{\Omega}^{G,d}$. It is easily checked that $z(\mathcal{P}) = \mathcal{N}$. $\square$

As an aside, the arbitrary choices made in this construction implies that Z is clearly not injective in general, or in other words, two partitions of different phylogenies can correspond to the same transmission tree. (In fact, some elements of $\mathbf{\Omega}$ cannot be produced by the construction of proposition S1.2.9 at all, for example, the bottom right example in figure 1 of the main text if $N = M = 3$.) The upshot of proposition S1.2.9 is that a MCMC procedure that fully explores the space of these partitioned phylogenies is also fully exploring the space of transmission trees amongst the elements of $\mathbf{A}$. We outline such a procedure in section S1.3.

So far, we have only dealt with the topology G of the phylogeny. If this construction is to be useful for epidemic reconstruction, branch lengths must also be considered. Let $\mathcal{P}$ be a partition of G, and suppose G is the topology of a phylogeny $\mathcal{G}$ with length function l and height function h. Suppose $a_i \in \mathbf{A}$ and that $z(\mathcal{P})(a_i) \neq \emptyset$. Let $u = e_{\mathcal{P}}(a_i)$ (the root of $H_{\mathcal{P},i}$, the subtree whose nodes comprise the

partition element corresponding to a_i), and let uP be the parent of u, which if it exists must be in a different partition element. An infection event occurs on the branch between uP and u, which means, assuming that internal nodes of G and transmissions do not occur at exactly the same time, that it occurs at a height in the open interval $(h(u), h(uP))$. It is more convenient to use a forwards timescale, so let $C : \mathbb{R} \to \mathbb{R}$ be a function converting between tree height and such a timescale (in the same units, so branch lengths are maintained). Let t_i^{inf} be the time, in the forwards scale, of this infection event. Let $q_i \in (0, 1)$ be such that $t_i^{\text{inf}} = C(h(uP)) + q_i l(u)$. If uP does not actually exist, i.e. a_i is the first host in the epidemic, then t_i^{inf} is between $C(h(r) + l(r))$ and $C(h(r))$ (remembering that we gave the root r of G a finite branch length) we can similarly define q_i such that $t_i^{\text{inf}} = C(h(r) + l(r)) + q_i l(r)$.

The combination of a phylogeny $\mathcal{G}$, map f from tip set to host set, partition $\mathcal{P}$ and a set of q_is for all elements $a_i \in \mathbf{A}$ then entirely determines the transmission history of the epidemic, describing which host infected which others and when. No assumptions are made at this, conceptual, level about when hosts cease to be infectious; a host can continue to infect others at any time following the time at which a sample was acquired from it. If, as will often be the case, this is an unreasonable assumption, the likelihood of such partitions can be evaluated to zero in the calculation of the posterior probability.

S1.3 MCMC procedure

We showed in section S1.2 that, if the sequence data is such that at least one sample is taken from each host, every transmission tree arises as at least one member of the set of partitioned phylogenies. Thus a Bayesian MCMC procedure to estimate time-resolved phylogenies can be extended to one that simultaneously samples from the probability distribution of reconstructed epidemics if each sampled tree $\mathcal{G}$ is augmented with a partition of its internal nodes as well as a set of values $\{q_i\}$ determining the exact times of infection. (An alternative approach, which we do not employ here but may be essential in extending the procedure to accommodate unsampled hosts, would be to insert an internal, binary node to represent each transmission event.) In this section we describe the MCMC moves developed

for this purpose in more detail than was given in the main text.

Note that these moves do not simultaneously change the value of any of the q_is, as new values of these are proposed and evaluated separately. Nevertheless, changes to either tree may involve resampling the times of infection of some hosts. If $a_i \in \mathbf{A}$, changing partition from $\mathcal{P}$ to $\mathcal{P}'$ may mean that $e_{\mathcal{P}}(a_i)$ and $e_{\mathcal{P}'}(a_i)$, the roots of the subtrees $H_{\mathcal{P},i}$ and $H_{\mathcal{P}',i}$ whose nodes make up the partition elements corresponding to a_i from $\mathcal{P}$ and $\mathcal{P}'$ respectively, are different nodes with different heights, and so while q_i will not change, the time of infection t_i^{inf} of a_i will. Even a move that has no effect on the partition or phylogenetic tree topology, such as a change to branch lengths, may also alter the height of $e_{\mathcal{P}}(a_i)$ and/or its parent, which will also modify t_i^{inf} while q_i remains fixed.

For the following definitions, recall that, for a host a_i and a partition $\mathcal{P}$ of a phylogenetic tree topology G, $H_{\mathcal{P},i}$ is the subtree of G whose nodes are mapped to a_i under $d_{\mathcal{P}}$, $e_{\mathcal{P}}(a_i)$ is the root of this subtree, and $c(a_i)$ is the MRCA of all tips corresponding to isolates sampled from a_i (which may be $e_{\mathcal{P}}(a_i)$ and otherwise is descended from it).

Definition S1.3.1. For a partition $\mathcal{P}$ of a phylogeny $\mathcal{G}$ with topology G determining a transmission tree on a set $\mathbf{A}$ of hosts, if u is a phylogenetic tree node with $d_{\mathcal{P}}(u) = a_i \in \mathbf{A}$ say u is *ancestral under* $\mathcal{P}$ if it is an ancestor of a node of the subtree $H_{\mathcal{P},i}$ which is also a tip of G. To put it another way, there is a tip v of G that is mapped to a_i by $d_{\mathcal{P}}$ such that it is possible to draw a simple path from v to the root of G that passes through u.

Definition S1.3.2. For a partition $\mathcal{P}$ of a phylogeny $\mathcal{G}$ with topology G determining a transmission tree on a set $\mathbf{A}$ of hosts, the *infection branch* for $a_i \in \mathbf{A}$ is the branch of G ending in $e_{\mathcal{P}}(a_i)$.

Definition S1.3.3. For a phylogeny $\mathcal{G}$ whose topology G is compatible with a map d taking each tip to the host of the corresponding isolate, say $a_i \in \mathbf{A}$ is *root-blocked* by $a_j \in \mathbf{A}$ if $c(a_j)$ is an ancestor of $c(a_i)$.

These definitions are illustrated in subfigure A of figure 2 of the main text. It should be noted that:

- For any valid partition $\mathcal{P}$, $d_{\mathcal{P}} \circ c(a_i)$ is a_i itself.
- If $N = M$, i.e. there is only one isolate per host, then $c(a_i)$ is the unique tip whose isolate was sampled from a_i for all i.
- As a result, if $N = M$ then no hosts are root-blocked by any others as all $c(a_i)$s are tips.
- If a_i is root-blocked by any a_j then the root r of G cannot be in the partition containing $d^{-1}(a_i)$, because $c(a_j)$ must lie on the path from $c(a_i)$ to r and for any $\mathcal{P}$, $d_{\mathcal{P}} \circ c(a_i) \neq d_{\mathcal{P}} \circ c(a_j)$ if $i \neq j$, so connectedness would be violated.

Suppose $\mathcal{G}$ is a phylogeny with tree topology G and $\mathcal{P} \in \mathbf{\Omega}^{G,d}$ a partition of its nodes according to definition S1.2.1. In what follows, trees are oriented so the "down" direction is towards the tips and the "up" towards the root.

S1.3.1 Infection branch operator

We randomly select a host a_i that is not the first host in the outbreak (i.e. $e_{\mathcal{P}}(a_i)$ is not the root of G). Let $u = e_{\mathcal{P}}(a_i)$ and uP be the parent of u (which must exist as we avoided the root). The operator performs both "downward" and "upward" moves, but if $u = c(a_i)$ (which is true if u is a tip) then the move must be upwards and if both a) $d_{\mathcal{P}}(u)$ is root-blocked by $d_{\mathcal{P}}(uP)$ and b) uP is ancestral under $\mathcal{P}$ then the move must be downwards; if both are true the move fails. In other cases, we select upwards or downwards each with probability 0.5. It must be that u and uP are in different elements of $\mathcal{P}$, and this implies that u is ancestral under $\mathcal{P}$ because the path from any node v that is not a descendant of u to u must pass through uP and if $d_{\mathcal{P}}(v) = a_i$ this would violate the connectedness requirement. Suppose $d_{\mathcal{P}}(uP) = a_j$.

Downward move We propose a new partition $\mathcal{P}'$ that has $d_{\mathcal{P}'}(u) = a_j$, moving the infection branch of a_i down the tree. Consider the two children uC_1 and uC_2 of u (as this is the downward move, u is

not a tip). At least one of these is mapped to the same element of $\mathbf{A}$ as u by $d_{\mathcal{P}}$ because u must be in the same element of $\mathcal{P}$ as $c \circ d_{\mathcal{P}}(u)$ and the path from u to this node in the subtree will intersect one of its children. If this is true of only one child then without loss of generality say it is uC_1. In this case we can simply make $\mathcal{P}'$ by setting $d_{\mathcal{P}'}(i) = a_j$ and leaving the rest of the partition unchanged; this is clearly still a valid partition because all subtrees remain connected. So suppose also $d_{\mathcal{P}}(uC_2) = d_{\mathcal{P}}(u)$. One and only one of uC_1 and uC_2 is ancestral under $\mathcal{P}$ (they would only both be if $u = c(a_i)$ which we prohibited and if neither is, the subgraph $H_{\mathcal{P},i}$ is either not connected or contains no tip) so, again without loss of generality, say it is uC_1. If we again set $d_{\mathcal{P}'}(u) = a_j$, the removal of u from $H_{\mathcal{P},i}$ splits the nodes of the latter into two sets, V_1 containing uC_1 and $c \circ d_{\mathcal{P}}(u)$, and V_2 containing uC_2 (and no tips). The nodes of both sets and the edges between them form connected subtrees of T, but their union is not connected. We complete the construction of $\mathcal{P}'$ by setting $d_{\mathcal{P}'}(v) = a_j$ for all $v \in V_2$. $H_{\mathcal{P}',i}$ and $H_{\mathcal{P}',j}$ are then connected.

The effect on the transmission tree is that all $a_k \in \mathbf{A}$ that have $z(\mathcal{P})(a_k) = a_i$ and $c(a_k)$ a descendant of (or equal to) uC_2 have $z(\mathcal{P}')(a_k) = a_j$ instead.

Upward move We propose a new partition $\mathcal{P}'$ that has $d_{\mathcal{P}'}(uP) = a_i$, moving the infection branch of a_i up the tree. We need to consider the grandparent uG of u if it exists, and its single sibling uS. At least one of uG and uS must be in the same element of $\mathcal{P}$ as uP (or else uP is not in a partition element containing a tip). If uG does not exist then this must be uS.

If $d_{\mathcal{P}}(uS) = a_j$ and either $d_{\mathcal{P}}(uG) \neq a_j$ or uG does not exist, then setting $d_{\mathcal{P}'}(uP) = a_i$ is all that is required to make $\mathcal{P}'$ a valid partition. The two or three nodes joined to uP by edges were all in different elements of $\mathcal{P}$ and remain so; uP was in the element of $\mathcal{P}$ containing one of its children and is moved to the one containing the other child in $\mathcal{P}'$. Similarly, if $d_{\mathcal{P}}(uG) = a_j$ and $d_{\mathcal{P}}(uS) \neq d_{\mathcal{P}}(uP)$, then all we need do is set $d_{\mathcal{P}'}(uP) = a_i$; the situation is the same except that the uP has moved from the element of $\mathcal{P}$ that contains its parent to one containing one of its children.

If uG exists and $d_{\mathcal{P}}(uS) = d_{\mathcal{P}}(uG) = a_j$, then the removal of uP from the subtree $H_{\mathcal{P},j}$ splits the

latter into two subtrees whose union is again not a connected subtree of G. Let the node sets of these two subtrees be V_1 and V_2, with V_1 containing uG and V_2 containing uS. V_1 and V_2 cannot both contain tips, because if they did, uP would be ancestral under $\mathcal{P}$ and $d_{\mathcal{P}}(u)$ would be root-blocked by $d_{\mathcal{P}}(uP)$ as $c(a_i)$ must be a descendant of u and $c(a_j)$ must be an ancestor of uP. If uP is ancestral under $\mathcal{P}$ then V_2 contains tips, and if it is not then V_1 does. We complete $\mathcal{P}'$ by setting $d_{\mathcal{P}'}(v) = a_i$ for all v in the set that contains no tips. $H_{\mathcal{P}',i}$ and $H_{\mathcal{P}',j}$ are now connected. Note that V_1 may contain the root node and if it does not contain $c(a_j)$ then the root's image under $d_{\mathcal{P}}$ is different from that under $d_{\mathcal{P}'}$, which is how this move may change the first host in the outbreak even though the root host is never chosen. This can be seen in subfigure C iv) of figure 2 of the main text.

If uP is not ancestral under $\mathcal{P}$, then the effect on the transmission tree is that all $a_k \in \mathbf{A}$ that have $z(\mathcal{P})(a_k) = a_j$ and $c(a_k)$ a descendant of (or equal to) uS have $z(\mathcal{P}')(a_k) = a_i$ instead. If uP is ancestral under $\mathcal{P}$ then, in $z(\mathcal{P}')$, a_i and a_j exchange infectors and all $a_k \in \mathbf{A}$ that have $z(\mathcal{P})(a_k) = a_j$ and $c(a_k)$ *not* a descendant of uS have $z(\mathcal{P}')(a_k) = a_i$ instead.

Hastings ratio We observe that:

- The downward move on u is reversed by the upward move on the child uC_1 of u that is ancestral under $\mathcal{P}$. The Hastings ratio is 2 if $uC_1 = c \circ d_{\mathcal{P}'}(uC_1)$, $\frac{1}{2}$ if uP is ancestral under $\mathcal{P}$ and $d_{\mathcal{P}}(u)$ is root-blocked by $d_{\mathcal{P}}(uP)$, and 1 if neither or both of these are true.

- If uP is not ancestral under $\mathcal{P}$, then the upward move on u is reversed by the downward move on uP. The ratio is $\frac{1}{2}$ if $u = c \circ d_{\mathcal{P}}(u)$, 2 if uG is ancestral under $\mathcal{P}$ and $d_{\mathcal{P}'}(uP)$ is root-blocked by $d_{\mathcal{P}'}(uG)$, and 1 if neither or both of these are true.

- If uP is ancestral under $\mathcal{P}$, and the upward move on u is possible, then it is reversed by the upward move on its sibling uS. The ratio is 2 if $uS = c \circ d_{\mathcal{P}'}(uS)$, $\frac{1}{2}$ if $u = c \circ d_{\mathcal{P}}(u)$, and 1 if neither or both of these are true.

S1.3.2 Phylogenetic tree operators

We have adapted the three standard tree moves used in BEAST (exchange, subtree slide, and Wilson-Balding [1, 2, 3]) such that they respect the transmission tree structure induced by partitioning the internal nodes. We give two versions of each:

- A "type A" operator which does not alter the transmission tree at all; all parental relationships remain the same.
- A "type B" operator which performs phylogenetic tree modifications which simultaneously rearrange the transmission tree by assigning new parents to one or two hosts.

For convenience, assume that the nodes of the phylogeny $\mathcal{G}$ are uniquely labelled. When $\mathcal{G}$ is modified to a proposed phylogeny $\mathcal{G}'$, it retains the same node set but has a different edge set. It is then meaningful for a single partition $\mathcal{P}$ to apply to the nodes of both $\mathcal{G}$ and $\mathcal{G}'$.

S1.3.2.1 Type A operators

Type A exchange Select a random node u that is not the root r of the phylogeny $\mathcal{G}$, and then randomly select a second node v, also not r and not the sibling uS of u, such that the parents uP and vP of u and v are in the same element of $\mathcal{P}$, $h(uP) > h(v)$, and $h(vP) > h(u)$ (recall that the height function is in backwards time from the last sample date). The last condition rules out the possibility that u is the ancestor of v or vice versa. If there is no such v then the operator fails. Otherwise, u and v exchange parents to obtain a proposed phylogenetic tree $\mathcal{G}'$ with the same partition of nodes $\mathcal{P}$. To see that $\mathcal{P}$ is still valid in terms of connectedness, note that the only nodes which are adjacent to different nodes before and after the move are u, uP, v, and vP. If anything has been disconnected it must have been along the branches connecting these. But if $d_{\mathcal{P}}(u) \neq d_{\mathcal{P}}(uP)$ then there was already a partition change along the branch from u to uP without the rules being violated, so if there is one on the branch that is now from u to vP then the rules still hold; no path from u to any other member of its

partition element has been modified. If, on the other hand, $d_{\mathcal{P}}(u) = d_{\mathcal{P}}(uP)$ then changing u's parent to vP means that it is still adjacent to a node with the same image under $d_{\mathcal{P}}$ as itself, and nothing has occurred to prevent there being a path between any two nodes in u's partition element. In both cases the same goes for v. The transmission tree structure is unchanged: if $d_{\mathcal{P}}(u) \neq d_{\mathcal{P}}(uP)$ then $d_{\mathcal{P}}(u)$ is infected by $d_{\mathcal{P}}(uP)$ before the move and by $d_{\mathcal{P}}(vP) = d_{\mathcal{P}}(uP)$ afterwards, whereas if $d_{\mathcal{P}}(u) = d_{\mathcal{P}}(uP)$ then $d_{\mathcal{P}}(u)$'s infection branch was not affected at all. Again, the same goes for v.

For the Hastings ratio, note that the partitioned tree obtained by selecting u and then v is exactly the same as that obtained by selecting v and then u. If u is selected first, let $n^{\mathrm{EA}}_{\mathcal{G},\mathcal{P}}(u)$ be the number of eligible nodes to be selected second (this is explicitly calculated every time the operator acts). The node u is selected first with probability $\frac{1}{2M-2}$ and then v is selected with probability $\frac{1}{n^{\mathrm{EA}}_{\mathcal{G},\mathcal{P}}(u)}$. The outcome is the same if v is selected first with probability $\frac{1}{2M-2}$ and then u with probability $\frac{1}{n^{\mathrm{EA}}_{\mathcal{G},\mathcal{P}}(v)}$. The denominator of the Hastings ratio is thus $\frac{1}{2M-2}\left(\frac{1}{n^{\mathrm{EA}}_{\mathcal{G},\mathcal{P}}(u)} + \frac{1}{n^{\mathrm{EA}}_{\mathcal{G},\mathcal{P}}(v)}\right)$. The move is reversed by selecting the same two nodes again (in either order), hence $n^{\mathrm{EA}}_{\mathcal{G}',\mathcal{P}}(u)$ and $n^{\mathrm{EA}}_{\mathcal{G}',\mathcal{P}}(v)$ are calculated and the ratio's numerator is $\frac{1}{2M-2}\left(\frac{1}{n^{\mathrm{EA}}_{\mathcal{G}',\mathcal{P}}(u)} + \frac{1}{n^{\mathrm{EA}}_{\mathcal{G}',\mathcal{P}}(v)}\right)$.

Type A subtree slide Select a random node u under the conditions that $u \neq r$ and at least one of u's grandparent uG or sibling uS is in the same element of $\mathcal{P}$ as its parent uP. Draw a distance $\Delta \in \mathbb{R}$ from some probability distribution that is symmetric about 0. The move aims to change the height of uP to $h(uP) + \Delta$. If $\Delta > 0$, find the node v amongst uS and its ancestors which has the minimum height while fulfilling $h(v) < h(uP) + \Delta$; this may be the root node or uS itself. If v is not in the same element of $\mathcal{P}$ as uP then the move fails. If $v = uS$ then simply change the height of uP to $h(uP) + \Delta$ and the topology is unchanged. Otherwise, modify the tree such that uP has height $h(uP) + \Delta$, parent vP (or no parent if $v = r$ in which case uP is now the root node) and child v, and uS has parent uG. Again, do not change $\mathcal{P}$. Connectedness rules are still obeyed because, in the new tree $\mathcal{G}'$, uP is adjacent to v, which is in the same element of $\mathcal{P}$ as itself. The transmission tree structure is unchanged as:

- The move does not change the partition, so no infection branch has changed if the corresponding phylogenetic tree branch was not modified by the move, except possibly by changing its length. This applies to the branch between u and uP as well as all branches adjacent to nodes other than u, uP, uG, uS, v, and vP.
- If uS and uP are in different elements of $\mathcal{P}$ then uP and uG are in the same one, so the infector of $d_{\mathcal{P}}(uS)$ remains the same.
- If uG and uP are in different elements of $\mathcal{P}$ then the move would have failed if $h(uP)+\Delta > h(uG)$ so the phylogenetic tree topology is unchanged.
- If v and vP are in different elements of $\mathcal{P}$ then uP, instead of v, is now the top end of $d_{\mathcal{P}}(uP)$'s infection branch, but $d_{\mathcal{P}}(uP) = d_{\mathcal{P}}(v)$ and its infector is still $d_{\mathcal{P}}(vP)$.

If $\Delta < 0$, then if $h(uP) + \Delta < h(u)$ the move fails. Otherwise, the move selects a node v at random with equal probability from the set W which consists of nodes w that:

1. Are descendants of uP but not descendants of u.
2. Have $h(w) < h(uP) + \Delta$ but $h(wP) > h(uP) + \Delta$; i.e. height $h(uP) + \Delta$ occurs along the branch which it terminates.
3. Have a parent in the same partition element as uP.

If W is empty the move fails. In the case that W consists only of uS then simply set $h(uP) = h(uP) + \Delta$ and the topology is unchanged. Otherwise, modify the tree such that uP has height $h(uP)+\Delta$, parent vP and child v, and uS has parent uG. Connectedness rules are still obeyed because there is an edge from uP to a node (vP) in the same element of the partition. The transmission tree structure is unchanged as:

- Again, the move does not change the partition, so any infection branches have not changed if the particular phylogenetic tree branch was not modified by the move, except by a change of length.
- If uS and uP are in different elements of $\mathcal{P}$ then the move would have failed if $h(uP)+\Delta < h(uS)$ so the topology is unchanged.
- If uG and uP are in different elements of $\mathcal{P}$ then uP and uS are in the same one, so the infector of $d_{\mathcal{P}}(uP)$ remains the same; uS is now the end of its infection branch.
- If v and vP are in different elements of $\mathcal{P}$ then the infector of $d_{\mathcal{P}}(v)$ is still $d_{\mathcal{P}}(vP) = d_{\mathcal{P}}(uP)$.

Suppose there are $n^{\text{SA}}_{\mathcal{G},\mathcal{P}}$ nodes eligible for this move before it occurs and $n^{\text{SA}}_{\mathcal{G}',\mathcal{P}}$ afterwards. If the topology did not change then the Hastings ratio is $\frac{n^{\text{SA}}_{\mathcal{G},\mathcal{P}}}{n^{\text{SA}}_{\mathcal{G}',\mathcal{P}}}$, which is 1 as such a move does not modify the set of possible candidate nodes. Otherwise, it is $\frac{|W|n^{\text{SA}}_{\mathcal{G},\mathcal{P}}}{n^{\text{SA}}_{\mathcal{G}',\mathcal{P}}}$ if $\Delta < 0$ and $\frac{n^{\text{SA}}_{\mathcal{G},\mathcal{P}}}{|W'|n^{\text{SA}}_{\mathcal{G}',\mathcal{P}}}$ if $\Delta > 0$, where W' is the set of nodes w that:

1. Are descendants of vP (in $\mathcal{G}$) but not descendants of u.
2. Have $h(w) < h(uP)$ but $h(wP) > h(uP)$.
3. Have $d_{\mathcal{P}}(wP) = d_{\mathcal{P}}(v)$.

Type A Wilson-Balding move Pick a node u under the same conditions as for the type A subtree slide: $u \neq r$ and at least one of u's grandparent uG and sibling uS is in the same element of $\mathcal{P}$ as its parent uP. Pick a second node v at random from amongst all nodes that are in the same element of $\mathcal{P}$ as uP, or whose parents are, and such that $h(vP) > h(u)$. The move fails if $uP = vP$, or $v = uP$. The node uP is pruned and reattached as a child of vP and the parent of v as with the standard Wilson-Balding move [1, 2]. As before, do not change $\mathcal{P}$. Connectedness rules are obeyed because there is an edge from uP to a node (either v or vP) in the same element of $\mathcal{P}$ as itself. The transmission tree structure is unchanged because if there was an infection event between uG and uC (and there was at

most one by construction) then there still is and it involves the same hosts, and likewise if there was one between vP and v then there still is and it involves the same hosts. If there was no infection event in either case then the removal or insertion of uP does not add one.

Notice that if u is subsequently selected for this move again, then the set of candidates for the second node is the same except that it excludes v but includes uS; in particular it has the same cardinality, as it did for the standard Wilson-Balding move. So only the choice of first node affects the Hastings ratio. It follows that this is the ratio from the standard Wilson-Balding move multiplied by $\frac{n^{\text{WA}}_{\mathcal{G},\mathcal{P}}}{n^{\text{WA}}_{\mathcal{G}',\mathcal{P}}}$, where $n^{\text{WA}}_{\mathcal{G},\mathcal{P}}$ is the number of nodes eligible for this move before it occurs and $n^{\text{WA}}_{\mathcal{G}',\mathcal{P}}$ is the number afterwards.

S1.3.2.2 Type B operators

Type B exchange Select a random node u, not r, whose parent uP is in a different element of $\mathcal{P}$ to itself. Pick a second node v, also not r and not uS, whose parent vP is also in a different element of $\mathcal{P}$ to itself (but this time the elements containing uP and vP do not have to be the same), such that $h(uP) > h(v)$, and $h(vP) > h(u)$, which as before prevents any ancestral relationship between u and v. If there is no such v then the operator fails. Otherwise, u and v exchange parents as with the type A operator to produce a proposal phylogeny $\mathcal{G}'$. $\mathcal{P}$ again does not change. That it preserves connectedness of subtrees is clear; it does not change where the boundaries between partition elements occur at all. The effect on the transmission tree is that $d_{\mathcal{P}}(u)$ and $d_{\mathcal{P}}(v)$ exchange parents (if their parents are different).

The Hastings ratio is calculated in effectively the same way as for the type A version, noting that the number of choices for u is just $N-1$. If $n^{\text{EB}}_{\mathcal{G},\mathcal{P}}(u)$ is the number of eligible choices for a second node if u is chosen first, then the ratio is $\left(\frac{1}{n^{\text{EB}}_{\mathcal{G}',\mathcal{P}}(u)} + \frac{1}{n^{\text{EB}}_{\mathcal{G}',\mathcal{P}}(v)}\right) / \left(\frac{1}{n^{\text{EB}}_{\mathcal{G},\mathcal{P}}(u)} + \frac{1}{n^{\text{EB}}_{\mathcal{G},\mathcal{P}}(v)}\right)$.

Type B subtree slide This time, u is a random node whose parent exists and is in a different element of $\mathcal{P}$ to itself. This implies that uP is in the same element as either uS or uG (if the latter exists) because otherwise uP would not be in a partition element containing a tip. The operator performs the

standard subtree slide move [3] on u, by drawing a $\Delta \in \mathbb{R}$ from a probability distribution symmetric around 0, finding a node v such that the height $h(uP) + \Delta$ occurs along the branch that v terminates, and inserting uP as the parent of v and (if v was not the root node) the child of vP. The state cannot, however, be left like this as there is no guarantee that uP is still adjacent to a node in the same partition element as itself. So $\mathcal{P}$ is changed to a new partition $\mathcal{P}'$ as follows: if vP does not exist or v and vP are in the same element of $\mathcal{P}$, uP is moved to the element containing v. Otherwise, it is moved to either the element containing v or that containing vP with equal probability. This reallocation is enough to ensure that $\mathcal{P}'$ obeys connectedness rules. The effect on the transmission tree is that $d_{\mathcal{P}}(u)$ is moved to become a child of either $d_{\mathcal{P}}(v)$ or $d_{\mathcal{P}}(vP)$. If $d_{\mathcal{P}}(uS) \neq d_{\mathcal{P}}(uG)$ then $d_{\mathcal{P}}(uS)$ was the child of $d_{\mathcal{P}}(uG)$ before the move and remains so.

Noting that there are always $N - 1$ choices for u, the Hastings ratio is the same as the standard subtree slide move, except that the denominator is multiplied by $\frac{1}{2}$ if vP exists and v and vP are not in the same element of $\mathcal{P}$, and the numerator is multiplied by $\frac{1}{2}$ if uG exists and uG and uS are not in the same element of $\mathcal{P}$.

Type B Wilson-Balding move In a similar way, u is randomly picked from the set of nodes whose parents exist and are in different subtrees to themselves, and the standard Wilson-Balding move is performed on it, inserting uP as a parent of another node v and a child of its parent if that exists. The reassignment of uP to a new subtree is performed in the same was as for type B subtree slide, and the adjustment to the Hastings ratio is identical. The effect on the transmission tree is also the same.

S1.3.3 Irreducibility of the chain

Suppose $\mathcal{P}$ is a partition of a phylogeny $\mathcal{G}$ with root node r and suppose $d_{\mathcal{P}}(r) = a_j$. We rely heavily on the fact that in the space of standard, unpartitioned phylogenies, the Wilson-Balding move on its own is sufficient for irreducibility [2]. Note that the following series of moves can transform a pair $\mathcal{G}, \mathcal{P}$ to a phylogeny in which, for each $a_i \in \mathbf{A}$, $c(a_i)$ and all its descendants are in the same partition element:

1. For any $a_i \in \mathbf{A}$, if $i \neq j$, a series of downward infection branch moves, starting with one on $e_{\mathcal{P}}(a_i)$, will eventually result in a partition $\mathcal{P}'$ in which $e_{\mathcal{P}'}(a_i) = c(a_i)$, in other words the earliest node u with $d_{\mathcal{P}}(u) = a_i$ is the most recent common ancestor of $d^{-1}(a_i)$.

2. As $c(a_i)$ now terminates the infection branch of a_i, the type B Wilson-Balding move can be used to make its parent $c(a_i)P$ the root node (if it is not already). After the move, $c(a_i)P$ will be in the same partition element as the old root node r.

3. Repeat this for all a_i with $i \neq j$.

Once this is completed, the result is a phylogeny and partition such that the only tips descended from $c(a_i)$ for each a_i (including a_j) are the members of the set $d^{-1}(a_i)$, and each $c(a_i)$ and all its descendants are in the same partition element. All nodes outside the clades rooted at each $c(a_i)$ are in the partition element containing $d^{-1}(a_j)$. In this tree, no host can root-block any other because there do not exist a_i and a_j with $c(a_i)$ an ancestor of $c(a_j)$ or vice versa. From this partition and phylogeny, for any $k \neq j$, a sequence of upward infection branch moves, starting with one on $c(a_k)$ and going up to the child of the root that is its ancestor, followed by a sequence of downwards moves starting with the child of the root that is not $c(a_k)$'s ancestor and going down to the parent of $c(a_j)$, will change the partition only by reassigning all nodes outside these clades to the element containing $d^{-1}(a_k)$.

If $\mathcal{G}$, $\mathcal{P}$ and $\mathcal{G}'$, $\mathcal{P}'$ are any two phylogeny-partition pairs such that the tips corresponding to the same isolate have the same height in both trees, each may be transformed into a tree and partition of the above form such that all the nodes that are not descendants of any $c(a_i)$ are in the partition element that contains $d^{-1}(a_j)$ for an arbitrary $a_j \in \mathbf{A}$. A combination of type A and B Wilson-Balding moves and branch length changes can then be used to transform one tree and partition of this form to another, with the type A operator handling topological modifications within each clade (as, if all nodes are in the same partition, the type A version is simply the standard Wilson-Balding move) and the type B moving the clades. An example is shown in figure S1.2. This shows irreducibility as all these moves are reversible.

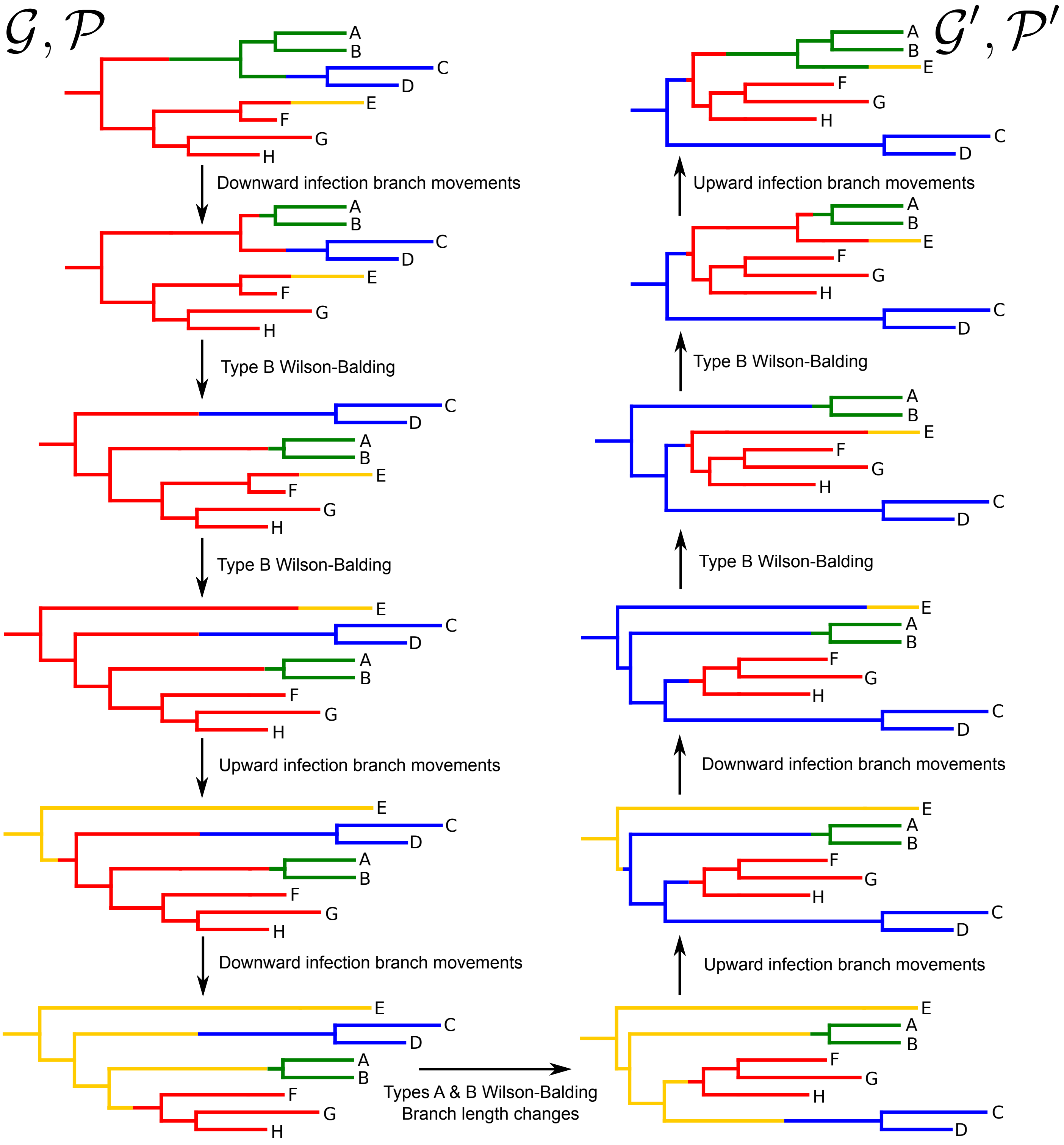

Figure S1.2: Illustration of the moves taking the phylogeny and partition $\mathcal{G}, \mathcal{P}$ (top left) to $\mathcal{G}', \mathcal{P}'$ (top right). Colours represent partition elements; tips correspond to isolates A to H.

References

[1] Wilson IJ, Balding DJ (1998) Genealogical inference from microsatellite data. Genetics 150: 499-510.

[2] Drummond AJ, Nicholls GK, Rodrigo AG, Solomon W (2002) Estimating mutation parameters, population history and genealogy simultaneously from temporally spaced sequence data. Genetics 161: 1307–1320.

[3] Hohna S, Defoin-Platel M, Drummond A (2008) Clock-constrained tree proposal operators in bayesian phylogenetic inference. In: 8th IEEE International Conference on BioInformatics and BioEngineering, 2008. BIBE 2008. pp. 1-7. doi:10.1109/BIBE.2008.4696663.
