# Supplementary material for: Epidemic Reconstruction in a Phylogenetics Framework: Transmission Trees as Partitions of the Node Set
Source: PLoS Comput Biol. 2015 Dec 30;11(12):e1004613. doi: 10.1371/journal.pcbi.1004613 (PMC4701012; doi:10.1371/journal.pcbi.1004613)
Supplement: S2 Text — Derivation of the probability p(N,Tinf,Ttrans,Tend|b,ϕ,ρ,λ,L). (PDF) [file pcbi.1004613.s005.pdf]

Epidemic Reconstruction in a Phylogenetics Framework: Transmission Trees as Partitions of the Node Set

Supplementary Methods 2

Matthew Hall Mark Woolhouse Andrew Rambaut

Derivation of $p(\mathcal{N}, \mathbf{T}^{\text{inf}}, \mathbf{T}^{\text{trans}}, \mathbf{T}^{\text{end}} | b, \phi, \rho, \lambda, L)$

We assume that for any a_i, the time of infectiousness t_i^{trans} is determined at t_i^{inf} and the time of noninfectiousness t_i^{end} is determined at t_i^{trans}. We now divide the timeline up into $2N$ intervals each ending in the infection or infectiousness time of a host that actually experienced infection, and introduce a set $\mathcal{E}(t)$ of those hosts which were infected but not infectious at time t. We have $2N$ real events and $2N-1$ intervals $I_2, \ldots, I_{2N}$ between them; we define I_1 as before. Suppose $t'_1 \ldots t'_{2N+N'}$ are the times of both infection and infectiousnesses sorted in increasing order, so I_i ends in t'_i for $2 \leq i \leq 2N$ and also define $I_{2N+1} = (t'_{2N}, t'_{2N+1}]$; the latter is from the last infectiousness time until the phantom infection time of all the non-infected susceptibles. Let $h(i)$ be the index of the host undergoing the event E_i at the end of I_i. If $i \leq 2N$ and I_i ends in an infection, E_i is taken to represent the combined occurrence of a) $a_{h(i)}$ being infected by $\mathcal{N}(a_{h(i)})$ at the end of I_i, b) the time of infectiousness of $a_{h(i)}$ being $t_{h(i)}^{\text{trans}}$, and c) no other infection events taking place during I_i. If, on the other hand, I_i ends in a host becoming infectious, E_i is the occurrence of a) the time of noninfectiousness of $a_{h(i)}$ being $t_{h(i)}^{\text{end}}$ and b) no infection events taking place during I_i. E_{2N+1} is the event that none of the remaining susceptibles were infected during I_{2N+1}.

We can now derive the probability $p(\mathcal{N}, \mathbf{T}^{\text{inf}}, \mathbf{T}^{\text{trans}}, \mathbf{T}^{\text{end}} | b, \phi, \rho, \lambda, L)$:

$$p(\mathcal{N}, \mathbf{T}^{\text{inf}}, \mathbf{T}^{\text{trans}}, \mathbf{T}^{\text{end}} | b, \phi, \rho, \lambda, L) = p(\{E_i : 1 \leq i \leq 2N+1\} | b, \phi, \rho, \lambda, L)$$
$$= \prod_{i=1}^{2N+1} p(E_i | \{E_j : j < i\}, b, \phi, \rho, \lambda, L). \quad (1)$$

If I_i ends in an infection, in other words $t'_i = t^{\text{inf}}_{h(i)}$, then if $i = 1$ we define a prior distribution for the time of the index infection and host of the index case as before. Otherwise, the calculation of $p(E_i | \{E_j : j < i\}, b, \phi, \rho, \lambda, L)$ can be performed much as before, differing only in the slightly modified notation. If $2 \leq i \leq 2N$,

- X_i is the probability that $\mathcal{N}(a_{h(i)})$ infected $a_{h(i)}$ at t'_i, but not before that during I_i:

$$X_i = bF(a_{h(i)}, \mathcal{N}(a_{h(i)})) \times \exp\left(-bF(a_{h(i)}, \mathcal{N}(a_{h(i)}))(t'_i - t'_{i-1})\right).$$

- Y_i is the probability that no host in $\mathcal{I}(t'_{i-1})$ other than $\mathcal{N}(a_{h(i)})$ infected $a_{h(i)}$ before t'_i in I_i. The upper bound on the time that such an a_j could have infected $a_{h(i)}$ before t'_i is still either t'_i itself if a_j was still infectious at that point or t^{end}_j if it was not. The term is:

$$Y_i = \prod_{\substack{a_j \in \mathcal{I}(t'_{i-1}) \\ a_j \neq \mathcal{N}(a_{h(i)})}} \exp\left(-bF(a_{h(i)}, a_j)(\min\{t'_i, t^{\text{end}}_j\} - t'_{i-1})\right).$$

- Z_i is the probability that no host in $\mathcal{I}(t'_{i-1})$ infected any host other than $a_{h(i)}$ in $\mathcal{S}(t'_{i-1})$ during I_i. Again, the upper bound on the time at which an a_j could infect a third host a_k before t'_i is $\min\{t'_i, t^{\text{end}}_j\}$.

$$Z_i = \prod_{a_j \in \mathcal{I}(t'_{i-1})} \prod_{\substack{a_k \in \mathcal{S}(t'_{i-1}) \\ k \neq h(i)}} \exp\left(-bF(a_j, a_k)(\min\{t'_i, t^{\text{end}}_j\} - t'_{i-1})\right).$$

Then $p(E_i | \{E_j : j < i\}, b, \phi, \rho, L) = X_i Y_i Z_i p(t^{\text{trans}}_{h(i)} | t^{\text{inf}}_{h(i)}, \lambda)$. Since λ are the actual lengths of each latent period rather than parameters of their distribution, and remembering that the length of the latent period of a_i is $lc(a_i)$, the last term in this product is trivially $\mathbb{1}_{t^{\text{trans}}_{h(i)} - t^{\text{inf}}_{h(i)} = lc(a_{h(i)})}$.

For the last interval, if $W = p(E_{2N+1}|\{E_i : i < 2N+1\}, b, \phi, \rho, L)$ then

$$W = \prod_{a_j \in \mathcal{I}(t'_{2N})} \prod_{a_k \in \mathcal{S}(t'_{2N})} \exp\left(-bF(a_j, a_k)(t_j^{\text{end}} - t'_{2N})\right).$$

If I_i ends in a host becoming infectious then $t'_i = t_{h(i)}^{\text{trans}}$. Now $p(E_i|\{E_j : j < i\}, b, \phi, \rho, L)$ is just $p(t_{h(i)}^{\text{end}}|t_{h(i)}^{\text{trans}}, \rho)$, the probability of the infectious period of $a_{h(i)}$, multiplied by

$$\prod_{a_j \in \mathcal{I}(t'_{i-1})} \prod_{a_k \in \mathcal{S}(t'_{i-1})} \exp\left(-bF(a_j, a_k)(\min\{t'_i, t_j^{\text{end}}\} - t'_{i-1})\right)$$

which is the probability that no susceptibles were infected on the interval. The product (1) can then be formed, and the same considerations regarding the distribution of infectious periods described for the SIR version in the main text apply.
